# Stratification of Volunteers According to Flavanone Metabolite Excretion and Phase II Metabolism Profile after Single Doses of ‘Pera’ Orange and ‘Moro’ Blood Orange Juices

**DOI:** 10.3390/nu13020473

**Published:** 2021-01-30

**Authors:** Alessandra Nishioka, Eric de Castro Tobaruela, Layanne Nascimento Fraga, Francisco A. Tomás-Barberán, Franco Maria Lajolo, Neuza Mariko Aymoto Hassimotto

**Affiliations:** 1 Food Research Center (FoRC) and School of Pharmaceutical Sciences, University of São Paulo, São Paulo 05508-000, Brazil; alessandranishioka@gmail.com (A.N.); erictobaruela@usp.br (E.d.C.T.); layannefraga@usp.br (L.N.F.); fmlajolo@usp.br (F.M.L.); 2 Research Group on Quality, Safety and Bioactivity of Plant Foods, Department of Food Science and Technology, CEBAS-CSIC, P.O. Box 164, Campus de Espinardo, 30100 Murcia, Spain; fatomas@cebas.csic.es

**Keywords:** citrus flavanones, bioavailability, interindividual variation, polymorphism, UDP-glucuronosyltransferase, gut microbiota

## Abstract

Large interindividual variations in the biological response to citrus flavanones have been observed, and this could be associated with high variations in their bioavailability. The aim of this study was to identify the main determinants underlying interindividual differences in citrus flavanone metabolism and excretion. In a randomized cross-over study, non-obese and obese volunteers, aged 19–40 years, ingested single doses of Pera and Moro orange juices, and urine was collected for 24 h. A large difference in the recovery of the urinary flavanone phase II metabolites was observed, with hesperetin-sulfate and hesperetin-sulfo-*O*-glucuronide being the major metabolites. Subjects were stratified according to their total excretion of flavanone metabolites as high, medium, and low excretors, but the expected correlation with the microbiome was not observed at the genus level. A second stratification was proposed according to phase II flavanone metabolism, whereby participants were divided into two excretion groups: Profiles A and B. Profile B individuals showed greater biotransformation of hesperetin-sulfate to hesperetin-sulfo-*O*-glucuronide, as well as transformation of flavanone-monoglucuronide to the respective diglucuronides, suggestive of an influence of polymorphisms on UDP-glucuronosyltransferase. In conclusion, this study proposes a new stratification of volunteers based on their metabolic profiles. Gut microbiota composition and polymorphisms of phase II enzymes may be related to the interindividual variability of metabolism.

## 1. Introduction

Orange fruit (*Citrus sinensis* L. Osbeck) and 100% orange juice, which are rich sources of flavanones, have been associated with health benefits leading to decreases in the occurrence of chronic non-communicable diseases such as obesity, diabetes, and hypertension [1,2,3,4]. The main flavanones present in these products are hesperidin (hesperetin-7-*O*-rutinoside) and narirutin (naringenin-7-*O*-rutinoside), which are biologically active in suppressing inflammation and oxidative stress as well as modulating several cell-signaling pathways [2,5,6,7,8]. Regarding the biological response, intake of 100% orange juice has been shown to have lipid- [9,10,11], glucose- [12], and blood-pressure-lowering activities as well as being associated with an improvement in insulin sensitivity [4,9,13,14].

Recent systematic reviews of animal studies and human clinical studies had different conclusions about citrus flavonoids and orange juice protection against cardiometabolic diseases. One of them had no definitive conclusion about the effects of orange juice intake or hesperidin on cardiovascular protection [3]. Another concluded that citrus flavonoids are promising candidates to protect against diabetes [15]. These contradictory findings are probably associated with the high heterogeneity in biological responses to phytochemicals and the food matrix, presenting high standard deviations, which reflect the interindividual variability.

With the aim of understanding the determinants of interindividual variation, the European Cooperation in Science and Technology (COST) Action POSITIVE proposed strategies to stratify the population into sub-sets, considering the main determinants in the bioavailability variation of plant food bioactive compounds, including genetic variability, the gut microbiome, and other factors, clustering individuals with similar metabolic phenotypes. Thus, the identification of individuals and biomarkers according to their metabotypes could be a way to identify those that are responsive to certain bioactive compounds [16,17,18].

The biological activity of flavanones depends on their absorption and circulating levels, and factors that affect their bioavailability could influence their biological activity. The absorption of the citrus flavanones naringenin- and hesperetin-rutinosides occurs in the large intestine, where the sugar moieties attached to the flavanone backbone are hydrolyzed by microbial α-rhamnosidases and β-glucosidases. The released flavanone aglycones are then absorbed and further conjugated with glucuronic acid and/or sulfate groups by the action of the phase II enzymes, UDP-glucuronosyltransferase (UGT), and sulfotransferase (SULT), respectively, reaching the circulation through the portal vein and undergoing further metabolization in the liver [19,20,21]. In this way, the gut microbiota is an important factor in citrus flavanone bioavailability [21,22,23,24,25,26,27,28].

Previous studies described the considerable variation in flavanone metabolite excretion after a single dose of orange juice, reflecting differences in bioavailability [29,30]. The significant heterogeneity observed may be related to several factors, such as gender, age, health condition, gene polymorphisms, anthropometric parameters, and the gut microbiota composition [18,21,28,29,31,32,33,34,35]. According to the urinary recovery of citrus flavanone metabolites, the clustering of subjects into groups with high, medium, and low urinary excretion capabilities [29,30] reinforces the influence of the gut microbiota in citrus flavanone metabolism. Furthermore, weak negative correlations with hesperetin excretion and age could also influence flavanone absorption after a single ingestion of orange juice [31]. However, no correlations with the subjects’ body mass index (BMI) and gender were observed [35].

Otherwise, subjects with higher BMI values excreted more 3-(3′-hydroxy-4′-methoxyphenyl) propanoic acid, a microbial catabolite of hesperetin, compared to those with lower BMI values [35], showing the importance of the microbial strains in flavanone metabolism. The effect of polymorphisms in phase II enzymes, including sulfotransferase (SULT) and UDP-glucuronosyltransferase (UGT), on flavonoid metabolism is less known but may be a driving factor in the variation in citrus flavanone metabolism. Genetic polymorphisms in the *SULT1A1* gene are associated with three alleles, showing differences in Vmax, with the SULT1A1*1 variant presenting the highest activity toward the flavonoids chrysin, genistein, and quercetin, followed by variants *3 and *2 [36]. Additionally, polymorphisms in the *UGT* gene, such as the UGT1A1*28 variant, present lower activity in the glucuronidation of endogenous substrates and xenobiotics [37]. In this way, there is still much to understand about the factors that influence flavanone bioavailability and affect its interindividual variation.

Considering that the identification and understanding of the main determinants underlying interindividual differences in the metabolism and excretion of citrus flavanones are essential to understand their bioactivity, this study evaluates the stratification of subjects by the excretion of flavanone phase II metabolites and the identification of flavanone excretion characteristics and, therefore, the exposure of sub-groups. For this purpose, obese and non-obese subjects ingested single doses of two orange juices obtained from the Pera (POJ) and Moro (MOJ) orange varieties. The Moro variety is known as the blood orange due to the presence of anthocyanin in its composition.

## 2. Material and Methods

### 2.1. Sampling of Orange Juices

Pasteurized orange juices obtained from *C. sinensis* L. cv Pera (POJ) and Moro (MOJ) were supplied by Fundecitrus (Araraquara, Brazil), located in the southeastern São Paulo state at 23°23′19″ S and 48°43′22″ W. All juices were filled into 1 L flasks and immediately stored at −20 °C.

### 2.2. Quantification of Soluble Sugar, Organic Acids, and Dietary Fiber

The soluble sugars were analyzed by high performance liquid chromatography (HPLC) coupled to a pulse amperometric detector according to Shiga et al. [38]. The organic acid contents were analyzed by HPLC in a HP1100 system (Hewlett-Packard Company, Palo Alto, CA, USA) coupled with a diode-array detector, equipped with a μBondpack C18 (300 mm × 3.6 mm i.d., Waters, Milford, MA, USA) and elution (flow rate of 0.5 mL.min^−1^) was carried out in isocratic conditions with 0.1% H_3_PO_4_, monitored at 210 nm. The content of total dietary fiber (TDF) and fractions were measured according to the method described by Association of Official Analytical Chemists (AOAC) (AOAC 991.43) [39].

### 2.3. Quantification of Flavanones in Orange Juice

The orange juices (10 mL) were centrifuged at 10,000× *g* for 15 min at 4 °C. The supernatant was eluted in a column of 1 g of polyamide (CC 6, Macherey–Nagel), previously preconditioned by passing methanol followed by deionized water. The phenolic compounds were eluted with methanol acidified with 2.5% acetic acid. The eluates were completely dried by rotary evaporation (Rotavapor, RE 120, Büchi, Flawil, Switzerland) under a vacuum at 40 °C, resuspended with methanol acidified with 5% acetic acid, and filtered through a 0.45 µm PVDF Millex filter (Millipore Ltd.a., Bedford, MA, USA) before HPLC analysis. The pellet was added to 20 mL of dimethyl sulfoxide, homogenized overnight at room temperature, centrifuged, and filtered through a 0.45 µm PVDF filter.

Samples were analyzed by HPLC on Agilent 2100 equipment coupled to a diode array detector (DAD) using a Prodigy 5 µm ODS3 column (250 × 4.60 mm) (Phenomenex Ltd., Cheshire, UK) with a flow rate of 1 mL.min^−1^ at 25 °C. Elution was carried out with a solvent gradient constituted of 0.5% formic acid in water (A) and 0.5% formic acid in acetonitrile (B). The solvent concentration gradient applied was 8% B at the beginning, 10% for 5 min, 17% for 10 min, 25% for 15 min, 50% for 25 min, 90% for 30 min, 50% for 32, and 8% for 35 min. The eluates were monitored at 280 and 525 nm [40]. Quantification was performed using a calibration curve of cyanidin-3-*O*-glucoside (at 525 nm) and hesperidin, narirutin, and didymin (Extrasynthese, Genay, France) at 280 nm.

Peak identification was carried out by Prominence liquid chromatography (Shimadzu, Japan) coupled to an ion trap mass spectrometer (Esquire HCT model, Bruker Daltonics, Billerica, MA, USA). The separation conditions were the same as those used for HPLC/DAD, and the flow rate was changed to 0.2 mL.min^−1^ to allow the eluate to pass through the mass spectrometer. The ESI was maintained in positive and negative modes for anthocyanins and other flavonoid classes, respectively. The mass operating conditions were programed to perform a full scan (*m*/*z* 100–1000), with a collision energy of 3000–3500 V, and a capillary temperature of 275 °C. Peak identification was carried out by the combined information provided by mass spectra, retention time, and literature data [41]. The identity was confirmed by co-elution with authentic standards.

### 2.4. Study Population

Twenty-seven women aged between 19 and 40 years were enrolled in this study. Seventeen subjects were non-obese (BMI = 19.00–24.99 kg.m^−2^) and 10 were obese (BMI ≥ 30.00 kg.m^−2^). Exclusion criteria applied were a history of gastrointestinal, liver, or kidney disease; habitual drinking or smoking; being diabetic or vegetarian; presenting with any type of infection; use of vitamin and mineral supplements, antibiotics, antacids, or medicines for diarrhea or constipation within four weeks before the start of the study; participation in intense physical activity; being pregnant; breastfeeding mothers; and individuals taking hormone therapy for menopause.

All procedures were approved by the Ethical Committee of the School of Pharmaceutical Science-University of São Paulo, São Paulo (CAAE 61707116.5.0000.0067) and were registered on the Brazilian Registry of Clinical Trials in www.ensaiosclinicos.gov.br (UTN: U1111-1216-2468). All participants were required to sign a written informed consent.

### 2.5. Study Design

The intervention study had a randomized cross-over design. Fifty-five volunteers were recruited, and only 27 met the inclusion criteria and completed both trials. Three days before and during the intervention, volunteers were instructed to avoid the consumption of citrus fruit, tomato, and fruits rich in anthocyanins, such as strawberries and grapes, and their derivatives, as well as tea and coffee. Each test phase comprised a single dose of each orange juice, POJ and MOJ, separated by a washout period of one week.

After 8 h of fasting, subjects ingested a single 600 mL dose of each juice, and urine was collected before intake and at time intervals of 0–4, 4–8, 8–12, and 12–24 h. The urine from volunteers who ingested MOJ was acidified with 50% formic acid. The total volume in each period was measured. Food restriction was requested during this period. Urine samples were stored in an ultra-freezer at −80 °C until analysis.

### 2.6. Intestinal Permeability Analysis

Three days before the test, subjects were advised to avoid fruits rich in mannitol, such as apple, guava, pear, grape, plum, cherry, apricot, raspberry, and blackberry. On the day of the trial, anthropometric variables (weight, height, waist circumference, % of body fat) were measured. Body fat (%) was measured using the Omron HBF-514C Digital Body Control Scale with Bioimpedance, and fat percentages of 9% to 22% and greater than or equal to 32% were used to classify subjects as non-obese or obese, respectively [42]. After 10 h of fasting, blood was collected in ethylenediaminetetraacetic acid (EDTA), fluoride, and non-anticoagulant tubes for biochemical analysis. Each subject collected their fecal samples at home using a sterile stool collector, and these were kept in a freezer at −20 °C and transported in appropriate containers with ice to the laboratory. Fecal samples were aliquoted immediately, without thawing, and stored at −80 °C.

Volunteers were asked to eliminate all urine and consume 200 mL of a solution containing 3 g of mannitol and 9.47 g of lactulose. Urine was collected within 5 h into a flask containing 1 mL of thimerosal (0.015 g). The total urine volume was recorded, and an aliquot was stored at −20 °C. All samples were filtered through a 0.45 µm PTFE Millex filter (Millipore Ltd.a., Bedford, MA, USA) before HPLC analysis.

HPLC analysis was performed using a Dionex ICS-5000^+^ DC detector (Thermo Fisher Scientific, Waltham, MA, USA) in a CarboPac PA1 column (4 × 250 mm) and an isocratic solvent of 500 mM NaOH at a flow rate of 1 mL.min^−1^. Quantification was performed using a calibration curve using mannitol and lactulose. The results are expressed as the lactulose/mannitol ratio (L/M).

### 2.7. Biochemical Parameters

Biochemical tests on the following compounds were performed by the clinical analysis laboratory of the University Hospital-University of São Paulo (HU-USP): glucose (enzymatic method—hexokinase); total cholesterol (colorimetric enzymatic method—cholesterol oxidase); low-density lipoprotein (LDL) cholesterol (obtained by the Friedewald equation); high-density lipoprotein (HDL) cholesterol (homogeneous colorimetric enzymatic method without precipitation); triglycerides (colorimetric enzymatic method—glycerol phosphate peroxidase according to Trinder); creatinine (colorimetric method—Jaffé); insulin (electrochemiluminescence—ECLIA); alanine aminotransferase (ALT) (enzymatic method—IFCC without pyridoxal phosphate); and aspartate aminotransferase (AST) (enzymatic method—modified IFCC). Homeostatic model assessment-insulin resistance (HOMA-IR) was calculated according to the formula (fasting insulin (µU.L^−1^) × fasting glucose (nmol.L^−1^))/22.5.

### 2.8. Plasma LPS and Zonulin

The plasma lipopolysaccharide (LPS) concentration was quantified using the QCL-1000^TM^ kit (Limulis Amebocyte Lysate, Lonza, Walkersville, MD, USA) in accordance with the manufacturer’s instructions. Zonulin was quantified using the IDK^®^ Zonulin ELISA K 5601 kit (Eagle Bioscience, NH, USA) in accordance with the manufacturer’s instructions.

### 2.9. Identification and Quantification of Urine Metabolites

Urine samples were centrifuged at 14,000× *g* for 5 min at 4 °C and filtered using a 0.22 µm PVDF filter (Millipore Ltd.a., Bedford, MA, USA). Samples were also analyzed by liquid chromatography UPLC-Nexera LC-30AD (Shimadzu, Kyoto, Japan) coupled to an EVOQ^TM^ triple quadrupole mass spectrometer (Bruker Daltonics). Separation of each metabolite was performed on a Poroshell 120 C18 column (2.7 µm, 100 × 3.0 mm) (Agilent, Palo Alto, CA, USA), equipped with a 20 × 4.0 mm guard column. The mobile phases used were as follows: (A) water/formic acid (99:1, *v*/*v*) and (B) acetonitrile. The solvent concentration gradient for B was 5% at the beginning, 18% at 7 min, 28% at 17 min, 50% at 17 min and 10 sec, 90% at 20 min, 90% at 20 min and 20 s, and 5% at 26 min a flow rate of 0.5 mL.min^−1^ at 25 °C. The eluates were monitored at 280 and 525 nm. The samples were analyzed in negative and positive modes at a source voltage of 3500 V, a cone temperature of 350 °C, a cone gas flow of 20 L.min^−1^, a heated probe temperature of 350 °C, a probe gas flow of 40 units, and a nebulizer gas flow of 50 units. Phase II metabolites were identified by the similarity of the mass spectra profile compared with the external standard of the metabolites naringenin-7-*O*-glucuronide, hesperetin-7-*O*-glucuronide, and hesperetin-3′-*O*-glucuronide, kindly donated by Dr. Kroon and Dr. Needs (Quadram Institute, Norwich, UK) and literature data. The area under the curve (AUC) of each peak was normalized by the value of creatinine to express the relative amount of flavanone metabolite excretion.

### 2.10. Gut Microbiota Profiling

Frozen fecal samples were sent for gut microbiota profile analysis by Neoprospecta Microbiome Technologies (Florianópolis, Brazil), DNA extraction was performed with the Illumina MiSeq platform (Illumina Inc., San Diego, CA, USA). The preparation followed the Bacterial NGS Sequence protocol [43]. The V3/V4 region of the 16S rRNA gene was amplified using primers 341F (CCTACGGGRSGCA-GCAG) and 806R (GGACTACHVGGGTWTCTAAT) [44,45]. DNA amplification was performed by a two-step PCR protocol, both in triplicate, using Platinum Taq Polymerase (Invitrogen, Carlsbad, USA) with the following conditions for PCR 1: 95 °C for 5 min, 25 cycles of 95 °C for 45 s, 55 °C for 30 s and 72 °C for 45 s, and a final extension of 72 °C for 2 min. For PCR 2, the protocol was as follows: 95 °C for 5 min, 10 cycles of 95 °C for 45 s, 66 °C for 30 s and 72 °C for 45 s, and a final extension of 72 °C for 2 min. The amplification result was purified with AMPureXP beads (Beckman Coulter, Brea, CA, USA). Library preparation (fixation of the TruSeq adapters and quantification of qPCR) was carried out using the Illumina 16S Library Preparation Protocol (Illumina Technical Note 15044223 Rev. B). Coverage of 100,000 reads was defined for each sample sequenced [46]. For taxonomic classification, operational taxonomic units (OTU) were matched against the SILVA Database [47].

### 2.11. Statistical Analysis

SPSS version 25.0 (SPSS, Inc., Chicago, IL, USA) was used to perform the statistical analysis. Data normality was checked using the Shapiro–Wilk’s test, and Levene’s test was used to assess the equality of variances. Nonparametric tests (Mann−Whitney and Kruskal–Wallis tests) were applied to assess differences in anthropometric and biochemical parameters, flavanone metabolites, and microbiota data between volunteer groups, and Spearman’s correlation coefficient was used to assess the associations between phase II flavanone metabolites and microbiota OTUs. Data are expressed as the mean ± standard error (SE), and differences were considered significant at *p* < 0.05. Multivariate analyses were performed using MetaboAnalyst 4.0 [48] after normalization by the median, log transformation, and Pareto scaling. Hierarchical cluster analysis was used to group volunteers according to their excretion profiles.

## 3. Results

### 3.1. Chemical Composition and Flavonoid Content of the Orange Juices and Biochemical and Anthropometric Profiles of the Volunteers

The chemical compositions of the orange juices are presented in Table S1. The TSS of POJ and MOJ was 7.2 and 6.5 °Brix, respectively. The TDF content was 0.18 and 0.31 g/100 mL for POJ and MOJ, respectively. The major flavanones in both orange juices, POJ and MOJ, were hesperidin and narirutin, followed by didymin (Table S2). The main anthocyanin identified in MOJ was cyanidin-3-*O*-glucoside, followed by cyanidin-*O*-malonyl-glucoside (Table S3), contributing to 35% of the total flavonoids. The flavanone concentration (hesperidin and narirutin) was similar in the POJ and MOJ orange juices (43.42 and 40.64 mg.100 mL^−1^, respectively). However, the soluble fraction of hesperidin was higher in MOJ than in POJ (16% and 9%, respectively) (Table S4).

Anthropometric variables, biochemical parameters, and intestinal permeability markers of both groups (obese and non-obese) are presented in Table 1. Total cholesterol and insulin concentrations and HOMA-IR values were found to be higher in the obese group as compared to the non-obese group (*p* < 0.05), and the volunteers from the obese group were diagnosed with insulin resistance according to the HOMA-IR assessment (3.02 ± 0.46).

### 3.2. Identification of Metabolites in the Urine after the Consumption of Orange Juice

Ten phase II metabolites of hesperetin (*m*/*z* 301) and naringenin (*m*/*z* 271) were identified in urine samples at different times after the consumption of both orange juices, including five hesperetin and five naringenin phase II metabolites identified as glucuronide and sulfate conjugates (Table 2). Hesperetin metabolites contributed to around 94–98% of the total metabolite recovery in the urine (Figure S1), mainly as sulfo-*O*-glucuronide conjugates (53–87%) (*m*/*z* 557), while naringenin was found as mono- and di-glucuronide conjugates (up to 99%) (*m*/*z* 447 and 623, respectively). The native flavanone-rutinosides and respective aglycone forms were not detected. Cyanidin metabolites were found in trace amounts in some urine samples of the volunteers who ingested MOJ.

### 3.3. Volunteer Stratification According to Total Excretion of Flavanone Metabolites

**Figure 1:**
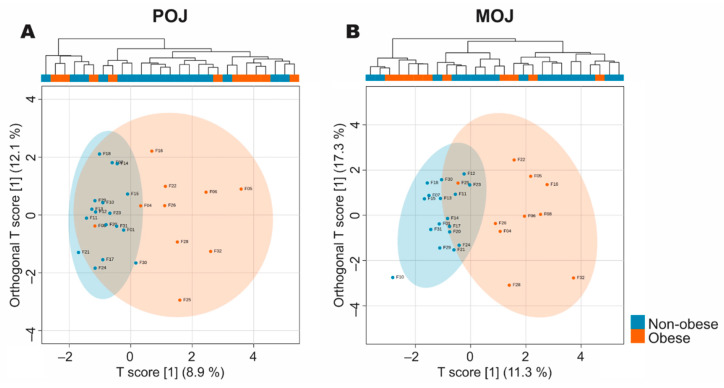
Orthogonal projections to latent structures discriminant analysis (oPLS-DA) of phase II flavanone metabolites excreted in urine 24 h (four collected intervals) after the consumption of (**A**) Pera orange juice (POJ) and (**B**) Moro orange juice (MOJ) in individuals with non-obese and obese BMI values (n = 17 non-obese and 10 obese volunteers).

Significant inter-individual variation in the excretion of phase II metabolites was observed among volunteers (Figure S2). Figure 1 shows the orthogonal projections to latent structures discriminant analysis (oPLS-DA) model of the two groups (obese and non-obese subjects) characterized in Table 1 as a function of the phase II urinary metabolites identified in the four collected intervals, totaling the first 24 h after both juices were consumed. The analysis showed that the members of the non-obese group had more similar metabolic profiles, while the obese group presented greater dispersion for both orange juices, POJ (Figure 1A) and MOJ (Figure 1B). The oPLSDA showed an BMI-based separation, however, the large variation among the profiles observed in the dendrogram of the cluster analysis, both intragroup and intergroup, suggests that in addition to BMI, other factors seem to determine the variation in metabolite excretion. High interindividual variation was also observed in the microbiome profile at the genus level, and the oPLS-DA model did not discriminate among groups according to BMI (Figure S3)

**Figure 2:**
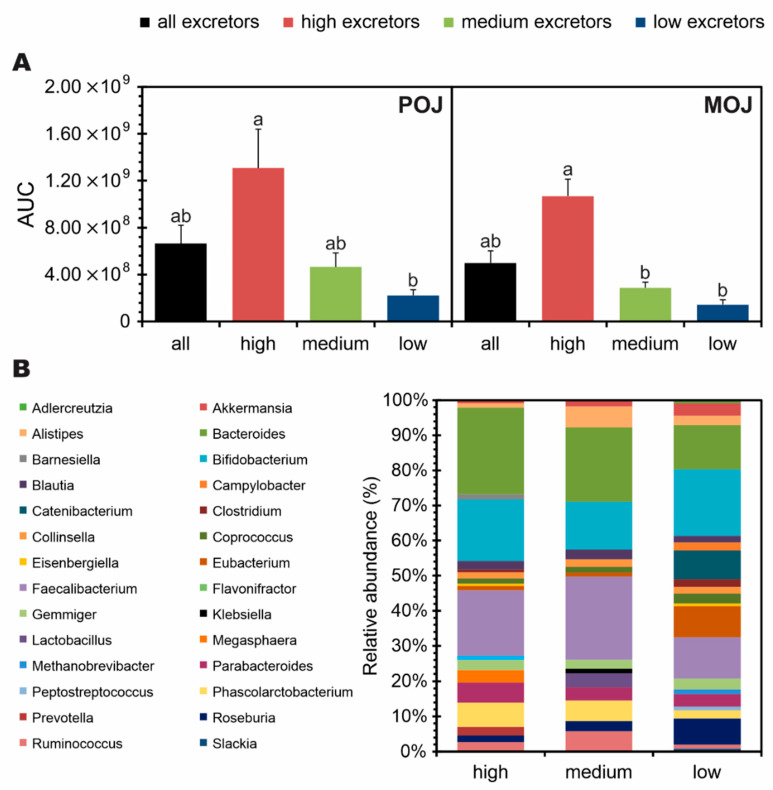
Stratification into high, medium, and low excretors, according to (**A**) total flavanone phase II metabolites excreted in urine 24 h after single doses of Pera (POJ) and Moro (MOJ) orange juices and to (**B**) the relative abundance of gut microbiota genus in the volunteers. Superscript letters indicate statistical significance among all, high, medium, and low excretors according to Kruskal-Wallis test (*p* < 0.05). AUC: Area Under the Curve.

In order to better understand and clarify the parameters that influenced the large variation observed, the volunteers were stratified into high, medium, and low excretors, according to the total excretion of flavanone metabolites (Figure 2A). Seven volunteers were characterized at each excretion level (n = 21). The volunteers classified as low excretors showed a significant difference in excretion compared with the high excretors, but medium excretors not differ from the other groups following consumption of POJ. In addition, a significant difference in excretion was observed between high and low excretors, but no difference was observed between medium and low excretors following consumption of MOJ. No differences in gut microbiota profiles were observed at the genus level among individuals with different excretion profiles (Figure 2B).

### 3.4. Stratification of Volunteers According to Phase II Metabolism

**Figure 3:**
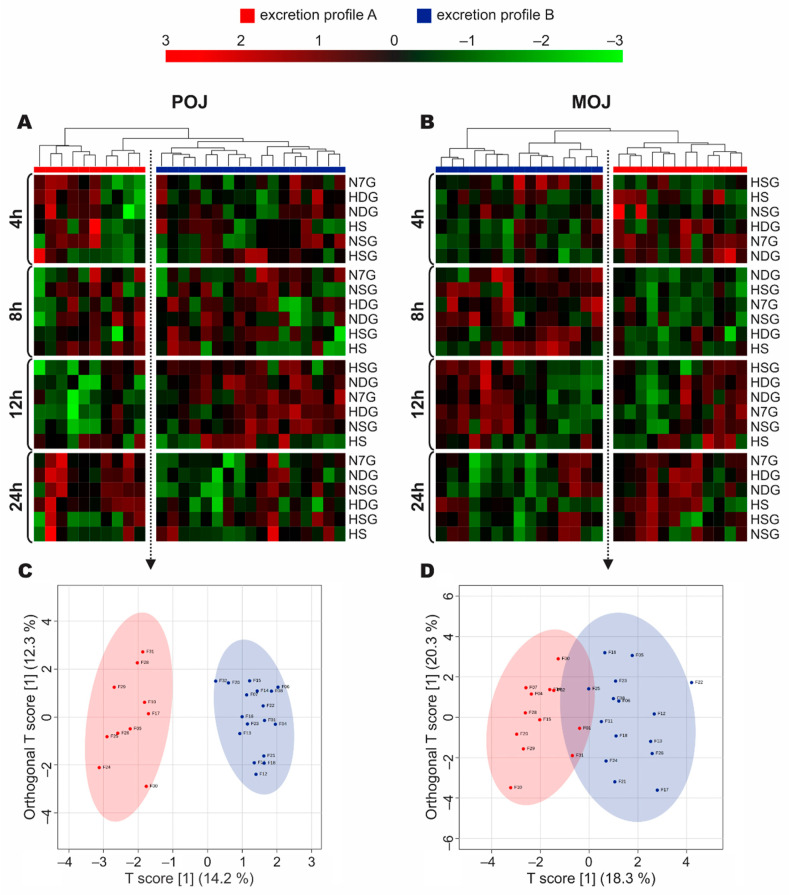
Heatmap of phase II flavanone metabolites excreted in urine over a 24 h period (four collected intervals) after the intake of Pera (POJ) and Moro (MOJ) orange juices ((**A**,**B**), respectively), separated into Excretion Profiles A and B. The oPLS-DA data show the Excretion Profiles according to phase II metabolite excretion in urine over the 24 h period ((**C**,**D**), respectively) (n of POJ = 10 volunteers with Profile A and 17 with Profile B; n of MOJ = 12 volunteers with Profile A and 15 with Profile B).

The 27 volunteers were distributed into two clusters, which were different from the previous ones, named ‘Excretion Profile A’ and ‘Excretion Profile B’. These were selected according to metabolic profiles after the intake of POJ (Figure 3A) and MOJ (Figure 3B). Through the Heatmap graph, we observed the separation of the two clusters, and their profiles had different responses over time, according to the excretion interval. Table S5 shows the anthropometric variables, biochemical parameters, and intestinal permeability markers of the volunteers in the new Excretion Profile groups. There were no significant differences between Profiles A and B, indicating that the new stratification was based on phase II metabolite excretion.

The new sub-set of excretors was evidenced by oPLS-DA (Figure 3C,D), with Excretion Profiles A and B presenting a more evident difference after the consumption of POJ (Figure 3C) than MOJ (Figure 3D), which showed that three volunteers had intermediate profiles.

For POJ (Figure 3A), volunteers from Profile A (n = 10) presented less metabolite excretion after an intermediate period of time (8–12 h), and this increased at the end of the 24 h period as compared with participants classified as belonging to Profile B (n = 17), who showed a high level of excretion at 8–12 h and reduced excretion from 12 to 24 h. For MOJ (Figure 3B), the same pattern was observed, but it was anticipated to occur 4–8 h after juice intake, with low and high metabolite excretion levels in Profile A (n = 12) and Profile B (n = 15), respectively. This ratio was maintained over an intermediate time period (8–12 h) and changed in the interval from 12 to 24 h. The new proposed stratification based on flavanone metabolism was not able to discriminate among volunteers with different levels of flavanone urinary excretion (Figure S4), as large interindividual variation was observed among volunteers.

### 3.5. Time-Dependent Excretion Curve According to Excretion Profile

**Figure 4:**
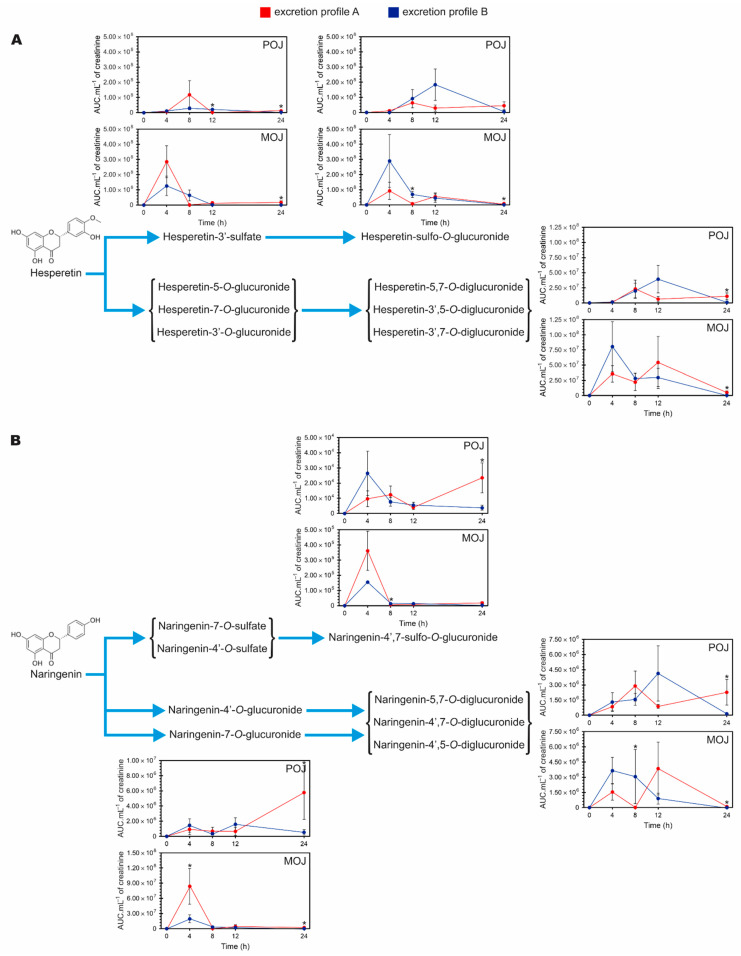
Time course curve of excretion of phase II flavanone metabolites in urine over a 24 h period for individuals classified as having Excretion Profiles A and B after consumption of Pera (POJ) and Moro (MOJ) orange juices: (**A**) Hesperetin metabolites and (**B**) naringenin metabolites. Number of participants: Profile A (n = 10) and Profile B (n = 17) for POJ; Profile A (n = 12) and Profile B (n = 15) for MOJ. Asterisks indicate statistical significance between excretion profiles A and B according to Mann–Whitney test (*p* < 0.05). AUC: Area Under the Curve.

Figure 4 shows the excretion curve over time for hesperetin (Figure 4A) and naringenin metabolites (Figure 4B), based on the previously classified profiles shown in Figure 3.

Excretion Profile A presents excretion peaks for hesperetin-sulfate, hesperetin-sulfo-O-glucuronide, and hesperetin-diglucuronide in the 4–8 h period after POJ intake. There was a decrease, followed by a slight but significant increase in excretion between 12 and 24 h after POJ intake (*p* = 0.05 and 0.003, respectively) (Table S6) as compared with Profile B. On the other hand, individuals with Profile B excreted more hesperetin-sulfo-O-glucuronide and -diglucuronide, with a shift in the peak of excretion (8–12 h) and lower excretion of hesperetin-sulfate (Figure 4A). The three hesperetin metabolites showed similar Excretion Profiles after the consumption of POJ and MOJ, but an earlier peak of excretion (0–4 h) was observed when MOJ was ingested for both Excretion Profiles, demonstrating that hesperetin from MOJ is metabolized faster by the gut microbiota than POJ. Also, a slight increase in excretion in the interval 12–24 h (excretion for Profile A individuals after MOJ intake) was observed (*p* = 0.039, 0.035, and 0.029, respectively. Table S6).

For individuals with Excretion Profile A, after the consumption of POJ, the naringenin metabolites (naringenin-sulfo-O-glucuronide, naringenin-7-O-glucuronide and -diglucuronide) were excreted continuously without a characteristic excretion peak. A significant increase in excretion occurred 12–24 h after juice consumption as compared with individuals with Profile B (*p* = 0.01, 0.035, and 0.007, respectively) (Figure 4B, Table S6). On the contrary, volunteers classified as having Profile B showed an excretion peak for naringenin-diglucuronide 8–12 h after POJ intake and then less excretion until 24 h. After MOJ intake, volunteers with both Profiles (A and B), showed naringenin-7-*O*-glucuronide and -sulfo-O-glucuronide excretion peaks at 0–4 h, but these were higher for individuals with Profile A than for those with Profile B. Naringenin-diglucuronide presented excretion peaks at 0–8 h and 8–12 h after MOJ consumption.

### 3.6. Accumulation of Excreted Metabolites Over a 24 h Period

**Figure 5:**
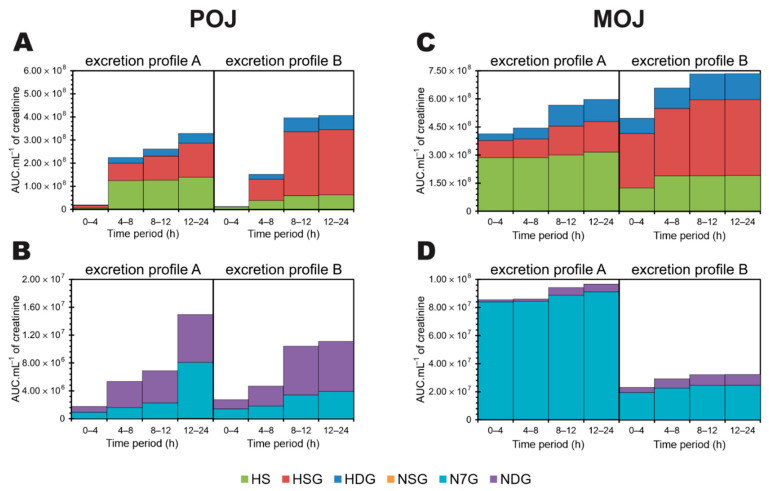
Recovery of urinary flavanone phase II metabolites over a 24-h period after single doses of Pera (POJ) (**A**,**B**) and Moro (MOJ) orange juices (**C**,**D**) for individuals classified as having Excretion Profiles A and B. Hesperetin metabolites (**A**,**C**) and naringenin metabolites (**B**,**D**) are shown (n of POJ = 10 volunteers with Profile A and 17 with Profile B; n of MOJ = 12 volunteers with Profile A and 15 with Profile B). HS: hesperetin-sulfate; HDG: hesperetin-diglucuronide; HSG: hesperetin-sulfo-*O*-glucuronide; NDG: naringenin-diglucuronide; NSG: naringenin-sulfo-*O*-glucuronide; N7G: naringenin-7-*O*-glucuronide. AUC: Area Under the Curve.

Figure 5 shows the recovery of urinary flavanone metabolites over a 24 h period after POJ and MOJ consumption. Flavanones from MOJ are more bioavailable than those from POJ, so a larger amount of hesperetin metabolites were recovered earlier (0–4 h) after MOJ intake (Figure 5A,C).

More hesperetin-sulfo-*O*-glucuronide was recovered from individuals with Excretion Profile B than from those with Excretion Profile A after the intake of POJ (around 60–70% and 33–45%, respectively) and MOJ (55–58% and 22–27%, respectively) throughout the 24 h period of collection (Figure 5A,C). In addition, the recovery of hesperetin metabolites occurred gradually over 24 h, starting from 0–4 h for individuals with Excretion Profile A, while the highest rate of recovery was observed later (8–12 h) for individuals with Profile B, reaching a plateau at 8–12 h (Profile A) or 12–24 h (Profile B) after POJ intake. Similar trends (8–24 h) were observed after MOJ intake (Figure 5C).

As for hesperetin, individuals with Excretion Profile A showed a gradual recovery of naringenin metabolites (Figure 5B,D) over the 24 h period. Unexpectedly, the urinary recovery of naringenin metabolites observed after MOJ intake was greater for individuals with Profile A than for those with profile B (Figure 5D). Furthermore, naringenin-7-*O*-glucuronide was the major naringenin metabolite recovered after MOJ intake (~97% and 75–80% for Profiles A and B, respectively). On the contrary, naringenin-diglucuronide was the major metabolite recovered after POJ intake (~50–70% and 47–67% for Profiles A and B, respectively).

**Figure 6:**
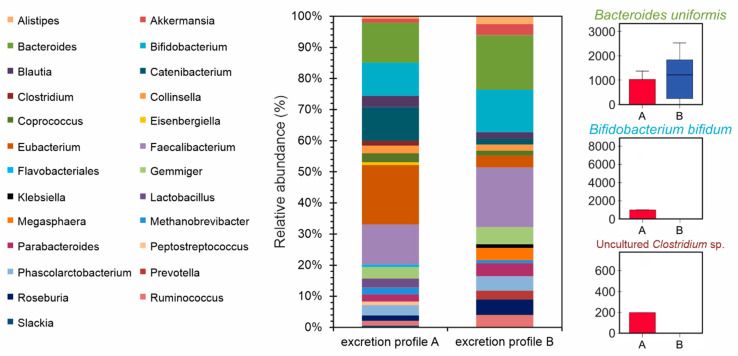
Relative abundance of gut microbiota genera in volunteers with Excretion Profiles A (n = 5) and B (n = 10).

Among the 27 volunteers, 15 volunteers shared similar excretion profiles after consuming POJ and MOJ. Based on their consistent excretion profiles, these volunteers were selected for microbiota analysis. Figure 6 shows the volunteers’ intestinal microbiota profiles according to their excretion profiles, providing evidence that individuals classified as having Profiles A and B have very distinct compositions in relation to the relative abundance of genera. The Mann−Whitney test was used to highlight significant differences at the genus and species levels (Table S7). A total of 25 genera and 46 species were identified using the SILVA database. Several bacterial genera that have already been associated with flavanone deglycosylation were detected, but no significant differences were observed between individuals with Excretion Profiles A and B. The species *Bacteroides uniformis* (*p* = 0.03) and *B. vulgatus* (*p* = 0.046) were detected in greater abundance in volunteers with Profile B than in those with Profile A. *Bifidobacterium bifidum* and an uncultured *Clostridium* species were only detected in volunteers with Excretion Profile A, and *Bifidobacterium longum* was found to be more abundant in volunteers with Profile A (*p* = 0.007) than in those with Profile B.

Table S7 shows the correlations among the metabolites excreted in the urine and the identified bacterial species. A positive correlation was found between hesperetin-sulfate and naringenin-sulfo-*O*-glucuronide excreted after POJ intake with the genus *Clostridium* and its uncultured species (0.537, *p* < 0.05). For MOJ, positive correlations were observed between naringenin-7-*O*-glucuronide and *Bacteroides uniformis* (0.564, *p* < 0.05) and between hesperetin-sulfo-*O*-glucuronide and *B. ovatus* (0.604, *p* < 0.05).

## 4. Discussion

Several studies have related orange juice consumption to various health benefits linked to the presence of the citrus flavanones narirutin, and hesperidin. However, the considerable interindividual variation in the biological response precluded the adequate interpretation of their efficacy [49]. The bioavailability of citrus flavanones is a key step in ensuring their bio-efficacy, and involves several phases, including food matrix release, absorption, metabolism, reaching the site of action, and excretion. Their absorption depends on extrinsic factors related to the food matrix and intrinsic factors such as age, gender, BMI, health status, and gut microbiota. These factors could be responsible for the significant heterogeneity in flavanone absorption and excretion [21,28,31,34,50,51].

For the first time, differences in the gut microbiota metabolism, which primarily influences the absorption of flavanones after hydrolysis, and further metabolic processes that degrade phenolics with the loss of the flavanone structure, as well as Phase II metabolism have been studied in a single intervention trial. In the present study, we stratified volunteers according to (1) the total excretion of flavanone metabolites to reflect the metabolism of the microbiota, and (2) the phase II metabolism profile in an attempt to explain the large considerable interindividual variation observed after the consumption of single doses of two orange juices.

Several metabolites, mainly mono and diglucuronides and sulfo and sulfo-glucuronides of naringenin and hesperetin were recovered in the urine after the intake of POJ and MOJ, showing that the aglycones were metabolized by phase II enzymes [34,37,51,52]. Hesperetin metabolites were found in the greatest abundance, mainly as sulfo and sulfo-glucuronide metabolites, while naringenin was found in minor amounts as mono and diglucuronide metabolites, showing the preference of sulfotransferase (SULTs) for hesperetin. Anthocyanin metabolites were detected in trace amounts in some but not all urine samples, although they are present in MOJ. This was expected due to the low bioavailability of anthocyanins, as previously reported [53,54,55].

Large interindividual differences in urinary phase II flavanone metabolites were observed after single doses of POJ and MOJ. Large variation in citrus flavanone absorption and excretion was also observed after the consumption of the orange juices, and this was used to stratify the volunteers into three levels of excretion, low, medium, and high flavanone excretors [29,30], suggesting the dependence of the specific microbiota to deglycosylate flavanone-rutinosides. In our study, the primary hypothesis was that subjects classified according to BMI (obese and non-obese) would have different and characteristic gut microbiome compositions. This could be a determining factor in the variation in flavanone excretion. However, no association was found between the BMI and gut microbiota profile data, and BMI and urinary flavanone excretion, which corroborates with the results found by Brett et al. [31]. In addition, the variability in the excretion of flavonoids was not correlated with anthropometric and clinical parameters or even with intestinal permeability.

The flavanone rutinosides hesperidin and narirutin are metabolized and absorbed after reaching the colon, where bacteria with α-rhamnosidase and β-glucosidase activities remove the rhamnose and glucose moieties and the aglycones can be absorbed [20,21]. Previous studies suggested that interindividual variation in flavanone bioavailability could reflect the diversity of several microbial strains with different degrees of deglycosylation efficiency [29,31,32,33,34,35,56]. In this way, gut microbiota modulation can improve the bioavailability of citrus flavanones, as demonstrated after chronic intake of a specific probiotic, *Bifidobacterium longum* R0175 [57]. Eight genera with α-rhamnosidase and β-glycosidase activity are involved in the metabolism of flavanones, including *Bifidobacterium*, *Adlercreutzia*, *Clostridium*, *Eubacterium*, *Bacteroides*, *Lactobacillus*, *Flavonifractor*, and *Parabacteroides* [58,59]. Some studies have identified species involved in the of flavanone metabolization, including *Bifidobacterium adolescentis, B. longum, B. catenulatum*, *B. dentium, B. breve, Enterococcus faecalis, Bacteroides ovatus, B. uniformis, Parabacteroides distasonis, Lactobacillus acidophilus, L. plantarum, Eubacterium ramulus, and Flavonifractor plautii* [58,59].

Thus, considering that the first stage of flavanone metabolism is mediated by the gut microbiota metabolism, we stratified the volunteers into groups of high, medium, and low excretors according to the total amount of flavanones excreted in the urine. However, no significant correlation with the gut microbiota composition was found at the genus and/or species level. In this way, the background diet could interfere with the microbial profile, making its annotation and the prediction of microbial metabolism difficult [16]. The relatively small size of the population included in our study and the temporal variability of the microbiome could have contributed to the low level of correlation.

Taking a different approach, which did not disregard the influence of BMI or gut microbiome metabolism, we proposed a new stratification based on flavanone phase II metabolite excretion profiles, named excretion ‘Profile A’ and ‘Profile B’. This was shown to be more effective than using the BMI itself.

The pathway proposed for hesperetin conjugation involves an initial sulfation process in the intestine and a second glucuronidation process in the liver [21]. This is in agreement with the present results, as hesperetin-sulfate showed an excretion peak before hesperetin-sulfo-*O*-glucuronide. Considering the new stratification, Profile B showed a greater excretion rate than Profile A, with similar trends for hesperetin-diglucuronide and naringenin-diglucuronide, suggesting a faster second glucuronidation for individuals with Profile B than for those with Profile A.

Polyphenol glucuronidation is mediated by UGTs. The UGT1 and UGT2 families are the most important drug-conjugation enzymes, with the UGT1A1 subfamily, which is primarily expressed in the liver, being the polymorphic isoform with the greatest role in phenolic glucuronidation [60]. Among the major enzymes responsible for hesperetin glucuronidation, UGT1A3 only produces 7-*O*-glucuronide while UGT1A7 mainly 3′-*O*-glucuronide. Other enzymes, including UGT1A1, UGT1A8, and UGT1A9, also catalyze hesperetin glucuronidation [61]. Polymorphic variation in UGT1A1 contributes to the variability in PK parameters and the altered activity [62]. For example, UGT1A1 is responsible for the metabolism of bilirubin, and a polymorphic variant UGT1A1*28 results in reduced UGT activity and, consequently, unconjugated hyperbilirubinemia, a condition known as Gilbert’s syndrome [37,63].

In addition, a higher urinary mutagenicity index value was observed after the exposure of well-cooked red meat to subjects with UGT*28/*28 genotypes than after exposure to subjects with *1/*28 genotypes, with mutagens excreted in the free and non-deactivated forms [64]. The type and frequency of the UGT1A1*28 minor allele depends on the genetic and geographic backgrounds [63]. It is detected in 3% of Asians, ~15% of Europeans, and up to 35% of Africans, who are carriers of Gilbert´s syndrome. In addition, 53% of premenopausal women carry the UGT1A1*28 minor alleles, including 11 homozygotes [65]. The effect of UGT polymorphisms on flavonoid clearance is still limited. A positive correlation was observed between the UGT1A1*28 variant and urinary isoflavone glycitein–sulfate recovery, but no association was observed with its reduced appearance in isoflavone glucuronides as compared with non-polymorphic carriers [65], suggesting that there is competition between the two phase II metabolism pathways. Thus, the observed effect of polymorphism on other xenobiotics may suggest that flavonoids have similar effects. Thus, the occurrence of the UGT1A1 variant associated with lower enzyme activity in some subjects could explain the low rate of biotransformation of hesperetin-sulfate to hesperetin-sulfo-*O*-glucuronide in volunteers with Excretion Profile A, as well as the low rates of transformation of hesperetin and naringenin monoglucuronides to their respective diglucuronides. In addition, the higher relative percentage recovery of hesperetin-sulfo-*O*-glucuronide in volunteers with Excretion Profile B corroborates a possible involvement of the UGT gene polymorphism in flavanone phase II metabolism. A similar pattern was also observed for naringenin metabolites. The polymorphism on UGT1A1 may be beneficial in the context of reduced conjugation of flavanones and a slow excretion rate. Therefore, polymorphic variants of phase II enzymes, such as UDP-glucuronosyltransferase, may contribute to the variability in flavanone metabolism observed in our study. This may affect their excretion and, therefore, the duration of tissue and cell exposure and, consequently, their bioactivity. However, further genotyping for polymorphisms in UGT and SULT must be conducted to confirm our results.

In general, a similar pattern of excretion was observed after MOJ intake, and the classification into Excretion Profiles A and B also fitted well. However, earlier metabolite peaks were observed for most of the hesperetin and naringenin metabolites following the consumption of MOJ compared with after POJ, suggesting a food matrix influence. Despite both orange juices presenting similar concentrations of hesperidin and naringenin, a higher amount of hesperidin was found to be soluble in MOJ than in POJ (16% and 9%, respectively), suggesting that is more bio-accessible. Vallejo et al. [29] showed that the absorption of hesperidin decreases when the flavanones precipitate and pass to the insoluble fraction and that flavanone solubility correlates positively with citrus flavanone bioavailability in several orange beverages. However, to the best of our knowledge, no studies have evaluated the metabolism and bioavailability of flavanones from blood oranges.

It seems that Profiles A and B are related to phase II metabolism differences, and therefore, they do not correlate with the high, medium, and low excretion groups. This was corroborated by the fact that the volunteers were not sorted into the same two groups in the two stratifications. The profiles did not correlate with the gut microbiota composition either, as the gut microbiota do not affect phase II metabolism. However, *Bacteroides uniformis* and *Bifidobacterium bifidum* species are responsible for the *O*-deglycosylation of flavanones [58,59], and they were detected in greater abundance in volunteers with Excretion Profile B as compared to those with Profile A. Furthermore, an uncultured *Clostridium* sp. has been related to the fission of C-ring [58,59], and this was only detected in volunteers with Excretion Profile A.

The correlation data from the present study show a positive correlation between the genus *Clostridium* and the hesperetin-sulfate and naringenin-sulfo-*O*-glucuronide metabolites, as well as between naringenin-7-*O*-glucuronide and *Bacteroides uniforms* and between hesperetin-sulfo-*O*-glucuronide and *B. ovatus.* These genera and species are associated with *O*-deglycosylation and the C-ring fission of flavonoids [58,59]. It is known that *Bacteroides*, *Lactobacillus*, and *Bifidobacterium* have rhamnosidase activity and, therefore, the excretion levels (quantity) of hesperetin conjugates and naringenin conjugates could be associated with some of these strains. However, the correlation of specific Phase II metabolites with some of these genera is unclear.

## 5. Conclusions

In conclusion, this study provides the essential insight needed to understand the factors involved in the interindividual variability in citrus flavanone bioavailability. We proposed a stratification of volunteers according to Excretion Profiles and flavanone metabolism. Gut microbiota composition and BMI were not clearly defined as major determinants in the separation of subjects. In addition, the observed results suggest, for the first time, that polymorphisms of UDP-glucuronosyltransferase could be involved in flavanone metabolite profiles. Once other factors, including uptake from intestinal mucosa, and transport into the circulation as well as transport into the liver, could also be involved in the flavanone metabolization process, further studies need to be done to evaluate the influence of polymorphisms of phase II enzymes on flavanone metabolism and to relate the proposed subject stratification to improvements in cardiometabolic health biomarkers.

## Figures and Tables

**Table 1:** Anthropometric variables, biochemical parameters, and intestinal permeability markers of the non-obese and obese volunteers.

Variable	Non-Obese (n = 17)	Obese (n = 10)	*p*-Value
Age (years)	26.76 ± 1.53	30.80 ± 3.02	0.197
Body weight (kg)	**56.45 ± 1.85 ^b^**	**83.29 ± 3.22 ^a^**	**0.001**
BMI (kg.m^−2^)	**21.19 ± 0.49 ^b^**	**31.56 ± 0.88 ^a^**	**0.001**
Body fat (%)	**28.23 ± 1.36 ^b^**	**47.03 ± 1.74 ^a^**	**0.001**
Creatinine (mg.dL^−1^)	0.71 ± 0.03	0.74 ± 0.02	0.376
AST (U.L^−1^)	18.59 ± 3.45	21.80 ± 3.44	0.545
ALT (U.L^−1^)	18.59 ± 6.75	30.00 ± 11.34	0.364
Cholesterol (mg.dL^−1^)			
LDL	89.71 ± 4.29	123.60 ± 17.17	0.084
HDL	69.12 ± 2.57	67.50 ± 9.77	0.876
Total	**176.94 ± 5.73 ^b^**	**217.00 ± 17.85 ^a^**	**0.016**
Triglycerides (mg.dL^−1^)	93.88 ± 9.39	175.40 ± 44.57	0.104
Glucose (mg/dL)	85.76 ± 1.82	102.00 ± 14.25	0.287
Insulin (mU.L^−1^)	**7.65 ± 1.00 ^b^**	**12.30 ± 1.74 ^a^**	**0.019**
HOMA-IR	**1.64 ± 0.23 ^b^**	**3.02 ± 0.46 ^a^**	**0.018**
Zonulin (ng.mL^−1^)	32.63 ± 1.92	29.28 ± 2.25	0.280
LPS (EU.mL^−1^)	0.09 ± 0.04	0.05 ± 0.01	0.448
Intestinal permeability	0.02 ± 0.00	0.01 ± 0.01	0.699

BMI: Body mass index. AST: aspartate transaminase. ALT: alanine transaminase. LDL: low-density lipoprotein. HDL: high-density lipoprotein. HOMA-IR: homeostatic model assessment-Insulin resistance. LPS: lipopolysaccharides. *p*-values calculated using the Mann–Whitney test. Values in bold letters and different superscript letters indicate statistical significance (*p* < 0.05) between volunteer groups for a given anthropometric or clinical parameter (mean ± standard error).

**Table 2:** Mass spectra data of phase II metabolites of flavanones detected in urine by LC-qTOF-MS/MS after single doses of pasteurized Pera and Moro orange juice.

Compound	Peak	RT (min)	[M-H]^−^ (*m/z*)	MS/MS (*m/z*)
Naringenin-diglucuronide—isomer 1	1	6.5	623.1285	447.0976/271.0690
Hesperetin-diglucuronide—isomer 1	2	8.3	653.1352	477.1054/301.0735
Naringenin-diglucuronide—isomer 2	3	8.8	623.1274	447.1274/271.0624
Naringenin-sulfo-*O*-glucuronide	4	8.8	527.0433	271.0585
Hesperetin-diglucuronide—isomer 2	5	10.6	653.1362	477.1041/301/0721
Hesperetin-sulfo-*O*-glucuronide	6	10.6	557.0699	477.1118/301.0759
Naringenin-7-*O*-glucuronide *	7	13.5	447.0929	271.0621
Naringenin-sulfate	8	14.5	351.0180	271.0622
Hesperetin-glucuronide	9	15.0	477.1045	301.0728
Hesperetin-sulfate	10	15.9	381.0343	301.0790

RT: retention time. * Compound identity confirmed by co-elution with standard.

## Supplementary Materials

The following are available online at https://www.mdpi.com/2072-6643/13/2/473/s1, Table S1: Chemical composition of pasteurized Pera and Moro orange juices. Table S2: Identity and mass spectrometric properties of flavonoids of pasteurized Pera and Moro orange juices. Table S3: Identity and mass spectrometric properties of the anthocyanins of pasteurized Moro orange juice. Table S4: Flavonoid content in pasteurized Pera and Moro orange juices. Table S5. Anthropometric variables, biochemical parameters, and intestinal permeability markers of the volunteers classified as having Excretion Profiles A and B before the orange juice consumption. Table S6: Phase II metabolites of flavanones detected in urine by LC-ESI-MS/MS after single dose of pasteurized Pera and Moro orange juices. Table S7: Relative abundance of significant gut microbiota OTUs (genus and species) in volunteers classified as having Excretion Profiles A and B before the orange juice consumption. Figure S1. Phase II metabolites of flavanones recovered over a 24 h period after single doses of Pera (POJ) and Moro (MOJ) orange juices. Figure S2: Total phase II metabolites of flavanones recovered over a 24 h period after single doses of Pera (POJ) and Moro (MOJ) orange juices by each volunteer according to BMI. Figure S3: Gut microbiota of volunteers, according to BMI (non-obese or obese groups), before the consumption of the orange juices. Figure S4: Total flavanone metabolites urinary recovered in urine over a 24 h period for individuals classified as having Excretion Profiles A and B after consumption of Pera (POJ) and Moro (MOJ) orange juices.
