# Supplementary material for: Stratification of Volunteers According to Flavanone Metabolite Excretion and Phase II Metabolism Profile after Single Doses of ‘Pera’ Orange and ‘Moro’ Blood Orange Juices
Source: Nutrients. 2021 Jan 30;13(2):473. doi: 10.3390/nu13020473 (PMC7910827; doi:10.3390/nu13020473)
Supplement: Supplementary file 1 [file nutrients-13-00473-s001.zip › supplementary/HassimottoNMA_Supplementary materials.docx]

### SUPLEMENTARY MATERIAL

### Stratification of volunteers according to flavanone metabolite excretion and phase II metabolism profile after single doses of ‘Pera’ orange and ‘Moro’ blood orange juices

### Alessandra Nishioka^1,†^, Eric de Castro Tobaruela^1,†^, Layanne Nascimento Fraga^1,†^, Francisco A. Tomás-Barberán^2^, Franco Maria Lajolo^1^, Neuza Mariko Aymoto Hassimotto^1*^

^1^Food Research Center (FoRC) and School of Pharmaceutical Sciences, University of São Paulo, 05508-000, São Paulo, Brazil

^2^Research Group on Quality, Safety and Bioactivity of Plant Foods, Department of Food Science and Technology, CEBAS-CSIC, P.O. Box 164, 30100, Campus de Espinardo, Murcia, Spain

^*^ Corresponding author: Neuza Mariko Aymoto Hassimotto, aymoto@usp.br

† These authors have contributed equally to this work.

**Table S1.** Chemical composition of pasteurized Pera and Moro orange juices

|  | POJ | MOJ |
| --- | --- | --- |
| TSS (°Brix) | 7.2 | 6.5 |
| pH | 4.08 | 4.14 |
|  |  |  |
| Soluble sugars (g.100 mL^-1^) |  |  |
| Sucrose | 2.55 ± 0.03 | 2.69 ± 0.25 |
| Fructose | 1.10 ± 0.02 | 1.36 ± 0.08 |
| Glucose | 0.98 ± 0.02 | 1.25 ± 0.04 |
| Total | 4.63 ± 0.87 | 5.30 ± 0.80 |
|  |  |  |
| Organic Acids (mg.100 mL^-1^) |  |  |
| Citric acid | 940.94 ± 0.44 | 966.24 ± 0.42 |
| Malic Acid | 419.16 ± 0.20 | 309.71 ± 0.11 |
| Succinic Acid | 102.98 ± 0.00 | 113.30 ± 0.00 |
| Tartaric acid | N.D. | 53.48 ± 0.03 |
| Ascorbic acid | 28.84 ± 0.01 | 25.24 ± 0.04 |
|  |  |  |
| Dietary fiber (g.100 mL^-1^) |  |  |
| Insoluble dietary fiber | 0.05 ± 0.01 | 0.20 ± 0.04 |
| Soluble dietary fiber | 0.13 ± 0.02 | 0.11 ± 0.00 |
| Total dietary fiber | 0.18 | 0.31 |

POJ: Pera orange juice. MOJ: Moro orange juice. TSS: total soluble solids. N.D.: Not detected. Values expressed as mean ± standard deviation (n = 3).

**Table S2.** Identity and mass spectrometric properties of flavonoids of pasteurized Pera and Moro orange juices

| Compound | RT  (min) | [M–H]^-^  (*m/z)* | MS/MS fragment  ions (*m/z*) | POJ | MOJ |
| --- | --- | --- | --- | --- | --- |
| Apigenin-6,8-di-C-glucuronide (Vicenin-2)* | 8.1 | 593.39 | 473.35/353.28 | + | + |
| Quercetin-3-*O*-rutinoside (Rutin)* | 12.6 | 609.42 | 563.38/301.10 | + | + |
| Naringenin-7-*O*-rutinoside (Narirutin)* | 16.6 | 579.39 | 271.23 | + | + |
| Hesperitin-7-*O*-rutinoside (Hesperidin)* | 19.3 | 609.43 | 301.24 | + | + |
| Quercetin-3-*O*-glucoside* | 21.6 | 463.40 | 301.24 | N.D. | + |
| Isosakuranetin-7-*O*-rutinoside (Didymin)* | 24.6 | 593.96 | 285.24 | + | + |

*Compound co-eluted with external standard. RT: Retention time. POJ: Pera orange juice. MOJ: Moro orange juice. N.D.: Not detected.

**Table S3.** Identity and mass spectrometric properties of the anthocyanins of pasteurized Moro orange juice

| Compound | RT  (min) | λ_max_ (nm) | [M]^+^  (*m/z)* | MS/MS fragment  ions (*m/z*) |
| --- | --- | --- | --- | --- |
| Delphinidin-3-*O*-glucoside* | 11.6 | 523 | 465.18 | 303.08 |
| Cyanidin-3-*O*-galactoside | 13.0 |  | 449.20 | 287.07 |
| Cyanidin-3-*O*-glucoside* | 13.3 | 280/516 | 449.19 | 287.07 |
| Peonidin-3-*O*-glucoside* | 15.9 |  | 463.22 | 301.09 |
| Delphinidin-malonyl-glucoside | 16.2 |  | 551.17 | 303.09^a^ |
| Cyanidin-3-O-(6″-malonyl glucoside) | 18.1 | 280/517 | 535.17 | 449.19/287.07^a^ |
| Cyanidin-3-O-(6″-dioxyalyl glucoside) | 19.1 | 520 | 593.22 | 287.08^a^ |

*Compound co-eluted with external standard. RT: Retention time. ^a^ Structure identification based on LC-MSn analysis [1] and Hillebrand et al. [2].

**Table S4.** Flavonoid content in pasteurized Pera and Moro orange juices

|  | POJ | | |  | MOJ | | |
| --- | --- | --- | --- | --- | --- | --- | --- |
|  | Supernatant | Pellet | Total |  | Supernatant | Pellet | Total |
| Narirutin | 1.10 ± 0.38 | 2.79 ± 0.01 | 3.89 |  | 2.48 ± 0.16 | 1.71 ± 0.56 | 4.19 |
| Hesperidin | 3.44 ± 1.52 | 36.09 ± 0.11 | 39.53 |  | 5.89 ± 0.36 | 30.56 ± 7.84 | 36.45 |
| Didymin | 0.96 ± 0.24 | 2.08 ± 0.01 | 3.04 |  | 0.68 ± 0.09 | 1.22 ± 0.40 | 1.90 |
| Cyn-3-*O*-glu | N.D. | N.D. | N.D. |  | 12.23 ± 1.01 | N.D. | 12.23 |
| Cyn-3-*O*-(malonyl)glu | N.D. | N.D. | N.D. |  | 2.89 ± 0.66 | N.D. | 2.89 |
| Total flavonoids | 7.61 | 40.96 | 48.57 |  | 24.70 | 33.49 | 58.19 |

POJ: Pera orange juice. MOJ: Moro orange juice. Cyn-3-*O*-glu: cyanidin-3-*O*-glucoside. Cyn-3-*O*-(malonyl)glu: cyanidin-3-*O*-(6″-malonyl)glucoside. N.D.: Not detected. Results expressed as mg.100 mL^-1^ (mean ± standard error).

**Table S5.** Anthropometric variables, biochemical parameters, and intestinal permeability markers of the volunteers classified as having Excretion Profiles A and B before the orange juice consumption

|  | POJ | | |  | MOJ | | |  | Common volunteers | | |
| --- | --- | --- | --- | --- | --- | --- | --- | --- | --- | --- | --- |
|  | Profile A  (n = 10) | Profile B  (n = 17) | *p*-value |  | Profile A  (n = 12) | Profile B  (n = 15) | *p*-value |  | Profile A  (n = 5) | Profile B  (n = 10) | *p*-value |
| Age (year) | 30.30 ± 2.80 | 27.06 ± 1.71 | 0.303 |  | 29.58 ± 2.62 | 27.20 ± 1.71 | 0.438 |  | 33.60 ± 4.55 | 27.30 ± 2.19 | 0.177 |
| Body weight (kg) | 72.59 ± 5.66 | 62.74 ± 3.27 | 0.117 |  | 64.74 ± 3.22 | 67.71 ± 4.86 | 0.635 |  | 65.68 ± 5.22 | 61.81 ± 4.79 | 0.626 |
| BMI (kg.m^-2^) | 27.14 ± 1.85 | 23.80 ± 1.26 | 0.136 |  | 24.44 ± 1.39 | 25.51 ± 1.62 | 0.630 |  | 24.72 ± 2.13 | 23.49 ± 1.72 | 0.674 |
| Obese (n) | 4 | 6 | - |  | 3 | 7 | - |  | 1 | 4 | - |
| Non-obese (n) | 6 | 11 | - |  | 9 | 8 | - |  | 4 | 6 | - |
| Body fat (%) | 38.81 ± 3.08 | 33.06 ± 2.67 | 0.185 |  | 35.25 ± 2.24 | 35.15 ± 3.33 | 0.678 |  | 35.32 ± 3.46 | 31.57 ± 4.02 | 0.559 |
| Creatinine (mg.dL^-1^) | 0.70 ± 0.02 | 0.74 ± 0.03 | 0.361 |  | 0.69 ± 0.03 | 0.74 ± 0.03 | 0.179 |  | 0.67 ± 0.03 | 0.75 ± 0.04 | 0.181 |
| AST (U.L^-1^) | 17.70 ± 1.87 | 21.00 ± 3.83 | 0.534 |  | 21.17 ± 4.84 | 18.67 ± 2.43 | 0.628 |  | 18.40 ± 3.14 | 19.50 ± 3.51 | 0.844 |
| ALT (U.L^-1^) | 17.10 ± 3.54 | 26.18 ± 9.22 | 0.471 |  | 24.67 ± 9.57 | 21.33 ± 7.75 | 0.786 |  | 19.20 ± 6.55 | 24.50 ± 11.59 | 0.764 |
| Cholesterol (mg.dL^-1^) |  |  |  |  |  |  |  |  |  |  |  |
| LDL | 104.00 ± 17.99 | 101.24 ± 5.94 | 0.862 |  | 107.92 ± 15.47 | 97.73 ± 5.55 | 0.714 |  | 119.80 ± 35.73 | 102.50 ± 7.23 | 0.714 |
| HDL | 68.40 ± 4.11 | 68.59 ± 5.71 | 0.982 |  | 67.92 ± 3.18 | 69.00 ± 6.57 | 0.892 |  | 72.60 ± 5.52 | 71.40 ± 9.52 | 0.934 |
| Total | 189.60 ± 18.95 | 193.06 ± 7.51 | 0.844 |  | 193.75 ± 16.19 | 190.20 ± 7.88 | 0.835 |  | 205.80 ± 37.36 | 198.60 ± 10.07 | 0.809 |
| Triglycerides (mg.dL^-1^) | 133.10 ± 47.39 | 118.76 ± 12.31 | 0.718 |  | 128.75 ± 39.77 | 120.33 ± 12.98 | 0.828 |  | 161.20 ± 97.38 | 128.00 ± 17.98 | 0.294 |
| Glucose (mg/dL) | 101.50 ± 14.28 | 86.06 ± 1.90 | 0.597 |  | 97.33 ± 12.23 | 87.33 ± 1.41 | 0.372 |  | 117.20 ± 28.13 | 88.10 ± 1.97 | 0.883 |
| Insulin (mU.L^-1^) | 8.40 ± 1.10 | 9.94 ± 1.43 | 0.460 |  | 7.50 ± 1.24 | 10.87 ± 1.38 | 0.089 |  | 6.20 ± 1.02 | 11.00 ± 2.01 | 0.130 |
| HOMA-IR | 2.13 ± 0.41 | 2.16 ± 0.33 | 0.948 |  | 1.87 ± 0.41 | 2.37 ± 0.32 | 0.338 |  | 2.00 ± 0.79 | 2.43 ± 0.46 | 0.621 |
| Zonulin (ng.mL^-1^) | 31.70 ± 2.44 | 31.20 ± 1.91 | 0.875 |  | 34.26 ± 2.67 | 29.09 ± 1.40 | 0.196 |  | 31.55 ± 4.71 | 27.71 ± 1.70 | 0.358 |
| LPS (EU.mL^-1^) | 0.05 ± 0.01 | 0.09 ± 0.04 | 0.430 |  | 0.10 ± 0.06 | 0.06 ± 0.01 | 0.501 |  | 0.03 ± 0.01 | 0.06 ± 0.01 | 0.249 |
| Permeability | 0.02 ± 0.01 | 0.02 ± 0.00 | 0.988 |  | 0.02 ± 0.01 | 0.01 ± 0.00 | 0.044 |  | 0.02 ± 0.01 | 0.01 ± 0.01 | 0.264 |

POJ: Pera orange juice. MOJ: Moro orange juice. BMI: Body mass index. AST: Aspartate transaminase. ALT: Alanine transaminase. LDL: Low-density lipoprotein. HDL: High-density lipoprotein. HOMA-IR: Homeostatic model assessment-Insulin resistance. LPS: Lipopolysaccharides. *P*-value calculated using Mann-Whitney Test. Values in bold letters and different superscript letters indicate statistical significance (*p* < 0.05) between volunteer groups at each anthropometric or clinical parameter (mean ± standard error).

**Table S7.** Relative abundance of significant gut microbiota OTUs (genus and species) in volunteers classified as having Excretion Profiles A and B before the orange juice consumption

|  | Excretion profile | | *p*-value |
| --- | --- | --- | --- |
|  | A (n = 5) | B (n = 10) |  |
| Methanobrevibacter | 119.80 ± 267.88 | 21.70 ± 68.62 | 0.535 |
| *Methanobrevibacter smithii* | 119.80 ± 267.88 | 21.70 ± 68.62 | 0.535 |
| Akkermansia | 74.00 ± 165.47 | 161.80 ± 345.46 | 0.861 |
| *Akkermansia muciniphila* | 74.00 ± 165.47 | 161.80 ± 345.46 | 0.861 |
| Klebsiella | N.D. | 25.80 ± 81.59 | - |
| Klebsiella unclassified species | N.D. | 25.80 ± 81.59 | - |
| Bifidobacterium | 2980.80 ± 1486.82 | 3097.14 ± 1332.82 | 0.685 |
| ***Bifidobacterium longum*** | **458.60 ± 158.15^a^** | **100.90 ± 175.45^b^** | **0.007** |
| *Bifidobacterium bifidum* | 1616.00 ± 3085.99 | N.D. | - |
| Bifidobacterium unclassified species | 906.20 ± 191.46 | 3445.17 ± 1469.58 | 0.465 |
| Collinsella | 689.50 ± 239.50 | 445.14 ± 112.36 | 0.143 |
| *Collinsella aerofaciens* | 520.00 ± 70.00 | 445.14 ± 112.36 | 0.380 |
| *Collinsella tanakaei* | 67.80 ± 151.61 | N.D. | - |
| Slackia | 32.80 ± 73.34 | N.D. | - |
| *Slackia isoflavoniconvertens* | 32.80 ± 73.34 | N.D. | - |
| Clostridium | 177.40 ± 298.16 | N.D. | - |
| uncultured *Clostridium* sp. | 177.40 ± 298.16 | N.D. | - |
| Coprococcus | 793.33 ± 376.12 | 355.00 ± 67.86 | 0.513 |
| *Coprococcus catus* | 793.33 ± 376.12 | 355.00 ± 67.86 | 0.513 |
| Blautia | 1009.00 ± 259.19 | 465.71 ± 146.06 | 0.186 |
| *Blautia faecis* | 830.67 ± 302.25 | 420.00 ± 147.6 | 0.297 |
| Eisenbergiella | 51.00 ± 114.04 | N.D. | - |
| *Eisenbergiella tayi* | 51.00 ± 114.04 | N.D. | - |
| Eubacterium | 5346.50 ± 5101.50 | 866.50 ± 537.67 | 0.643 |
| *Eubacterium ramulus* | 5346.50 ± 5101.50 | 866.50 ± 537.67 | 0.643 |
| Roseburia | 483.50 ± 298.50 | 1119.17 ± 716.08 | 0.739 |
| *Roseburia inulinivorans* | 37.00 ± 82.73 | 184.10 ± 286.73 | 0.307 |
| *Roseburia hominis* | 156.40 ± 349.72 | 487.40 ± 1213.60 | 0.753 |
| Ruminococcus | 438.50 ± 195.50 | 912.00 ± 467.46 | 0.564 |
| *Ruminococcus bromii* | 126.80 ± 283.53 | 197.60 ± 473.61 | 0.901 |
| *Ruminococcus callidus* | N.D. | 38.20 ± 120.80 | - |
| *Ruminococcus faecis* | 48.60 ± 108.67 | 37.80 ± 119.53 | 0.679 |
| *Ruminococcus obeum* | 514.67 ± 304.22 | 290.00 ± 33.46 | 0.724 |
| Gemmiger | 1043.33 ± 324.48 | 1264.14 ± 651.72 | 0.425 |
| *Gemmiger formicilis* | 1043.33 ± 324.48 | 1264.14 ± 651.72 | 0.425 |
| Faecalibacterium | 3620.67 ± 2775.51 | 4346.78 ± 556.20 | 0.405 |
| *Faecalibacterium prausnitzii* | 3620.67 ± 2775.51 | 4346.78 ± 556.20 | 0.405 |
| Flavobacteriales | 38.20 ± 85.42 | N.D. | 0.157 |
| Flavobacteriales unclassified species | 38.20 ± 85.42 | N.D. | 0.157 |

**Table S7.** *Cont.*

|  | Excretion profile | | *p*-value |
| --- | --- | --- | --- |
|  | A (n = 5) | B (n = 10) |  |
| **Alistipes** | **41.40 ± 92.57^b^** | **562.60 ± 727.72^a^** | **0.039** |
| *Alistipes inops* | N.D. | 57.80 ± 182.78 | - |
| *Alistipes indistinctus* | N.D. | 26.60 ± 84.12 | - |
| *Alistipes shahii* | N.D. | 46.50 ± 98.65 | - |
| Alistipes unclassified species | 41.40 ± 92.57 | 431.70 ± 698.27 | 0.167 |
| Parabacteroides | 1080.00 ± 395.43 | 1210.00 ± 392.21 | 0.838 |
| *Parabacteroides distasonis* | 668.50 ± 373.50 | 1252.20 ± 381.46 | 0.439 |
| *Parabacteroides goldsteinii* | 163.20 ± 364.93 | 32.70 ± 103.41 | 0.535 |
| *Parabacteroides johnsonii* | N.D. | 19.30 ± 61.03 | - |
| *Parabacteroides merdae* | 543.50 ± 270.50 | 483.17 ± 175.33 | 0.399 |
| Prevotella | N.D. | 63.90 ± 202.07 | - |
| *Prevotella copri* | N.D. | 63.90 ± 202.07 | - |
| Bacteroides | 3590.25 ± 2174.24 | 3982.33 ± 762.50 | 0.440 |
| *Bacteroides massiliensis* | N.D. | 205.70 ± 463.23 | - |
| *Bacteroides ovatus* | 1995.60 ± 4462.30 | 257.90 ± 416.66 | 0.662 |
| *Bacteroides plebeius* | N.D. | 201.80 ± 434.47 | - |
| *Bacteroides stercoris* | N.D. | 192.20 ± 439.30 | - |
| ***Bacteroides uniformis*** | **479.60 ± 667.76^b^** | **1116.80 ± 871.24^a^** | **0.003** |
| ***Bacteroides vulgatus*** | **126.20 ± 207.52 ^b^** | **1440.00 ± 1394.90^a^** | **0.046** |
| *Bacteroides xylanisolvens* | 102.80 ± 229.87 | 169.70 ± 376.45 | 0.861 |
| Bacteroides unclassified species | 168.00 ± 375.66 | N.D. | - |
| Bacteroidetes | 61.20 ± 136.85 | N.D. | - |
| *Bacteroidetes bacterium* MU2 | 61.20 ± 136.85 | N.D. | - |
| Lactobacillus | 165.00 ± 368.95 | N.D. | - |
| *Lactobacillus ruminis* | 165.00 ± 368.95 | N.D. | - |
| Catenibacterium | 605.40 ± 1353.72 | 44.00 ± 139.14 | 0.535 |
| *Catenibacterium mitsuokai* | 605.40 ± 1353.72 | 44.00 ± 139.14 | 0.535 |
| Megasphaera | N.D. | 88.50 ± 279.86 | - |
| *Megasphaera elsdenii* | N.D. | 88.50 ± 279.86 | - |
| Phascolarctobacterium | 185.20 ± 414.12 | 533.40 ± 752.09 | 0.333 |
| *Phascolarctobacterium faecium* | 185.20 ± 414.12 | 533.40 ± 752.09 | 0.333 |
| Peptostreptococcus | 63.40 ± 141.77 | N.D. | - |
| *Peptostreptococcus anaerobius* | 63.40 ± 141.77 | N.D. | - |
| Unidentified | 58443.40 ± 9580.32 | 58308.20 ± 4083.44 | 0.806 |

OTU: Operational taxonomic unit. A: Excretion Profile A. B: Excretion Profile B. N.D.: Not detected. *P*-value calculated using Mann-Whitney Test. Values in bold letters and different superscript letters indicate statistical significance (*p* < 0.05) at the same OTU (mean ± standard error).


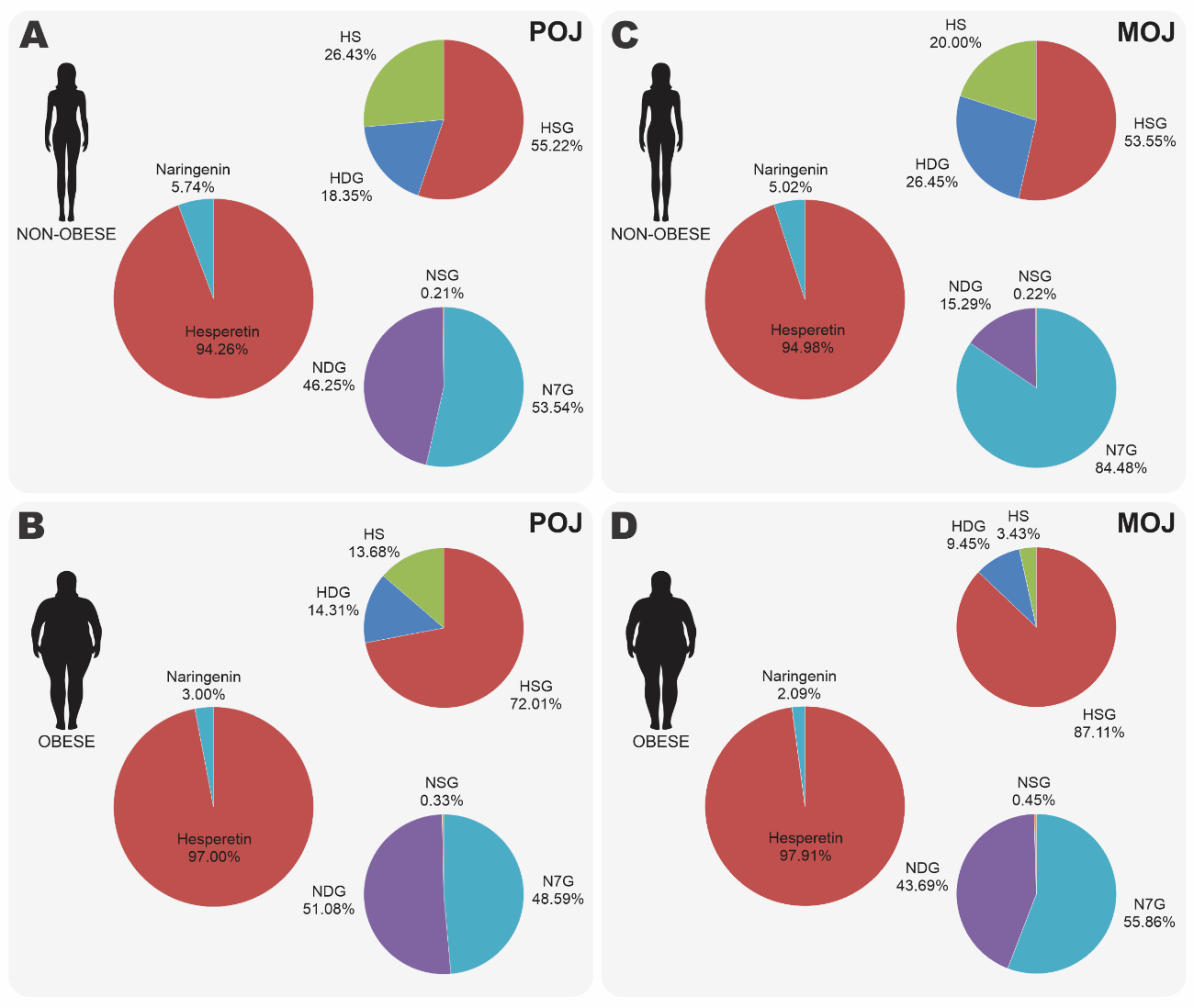
**Figure S1.** Phase II metabolites of flavanones recovered over a 24-hour period after single doses of Pera (POJ) and Moro (MOJ) orange juices. (A) Excretion of non-obese volunteers (n = 17) after POJ consumption. (B) Excretion of obese volunteers (n = 10) after POJ consumption. (C) Excretion of non-obese volunteers after MOJ consumption. (D) Excretion of obese volunteers after MOJ consumption. HS: Hesperetin-sulfate. HDG: Hesperetin-diglucuronide. HSG: Hesperetin-sulfo-*O*-glucuronide. NDG: Naringenin-diglucuronide. NSG: Naringenin-sulfo-*O*-glucuronide. N7G: Naringenin-7-*O*-glucuronide.

**
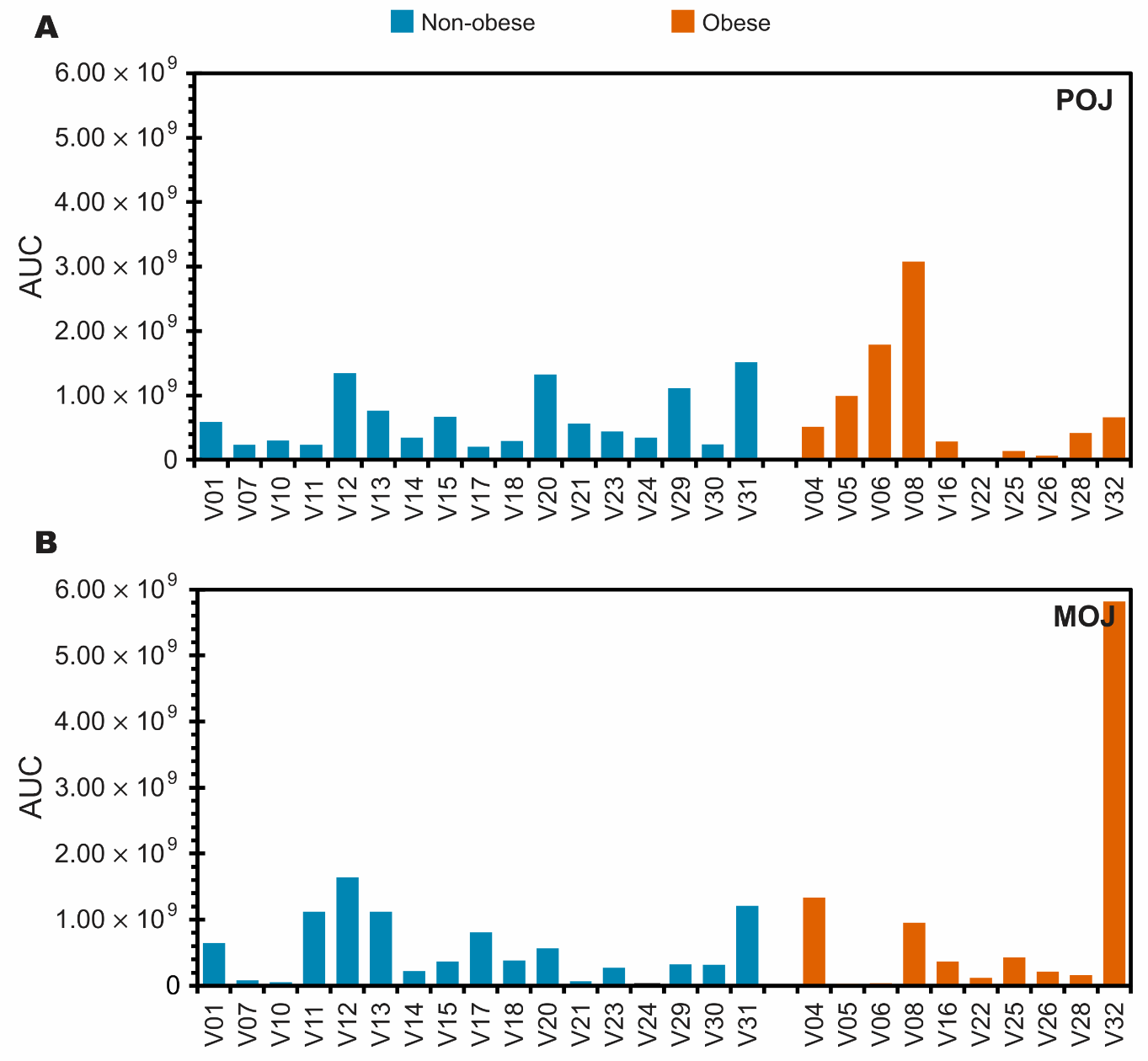
Figure S2.** Total phase II metabolites of flavanones recovered over a 24-hour period after single doses of Pera (POJ) and Moro (MOJ) orange juices by each volunteer according to BMI. (A) Excretion of non-obese (n = 17) and obese volunteers (n = 10) volunteers after POJ consumption. (B) Excretion of non-obese (n = 17) and obese volunteers (n = 10) volunteers after MOJ consumption. AUC: Area under the curve.


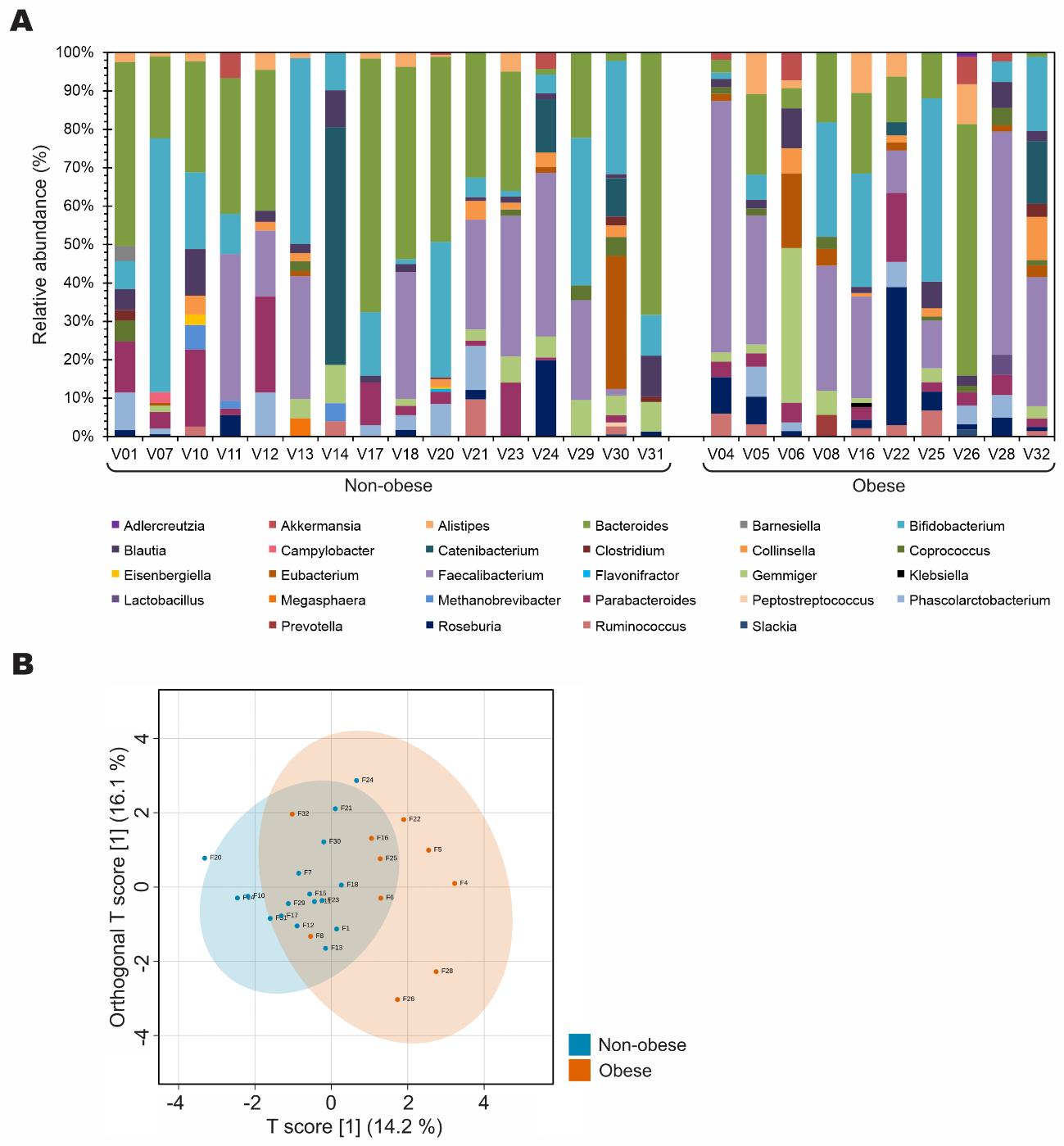


**Figure S3.** Gut microbiota of volunteers, according to BMI (non-obese or obese groups), before the consumption of the orange juices. (A) Relative abundance (%) of gut microbiota by genus to each volunteer. (B) oPLS-DA of volunteers as a function of gut microbiota genus. (n = 17 non-obese and 10 obese volunteers).


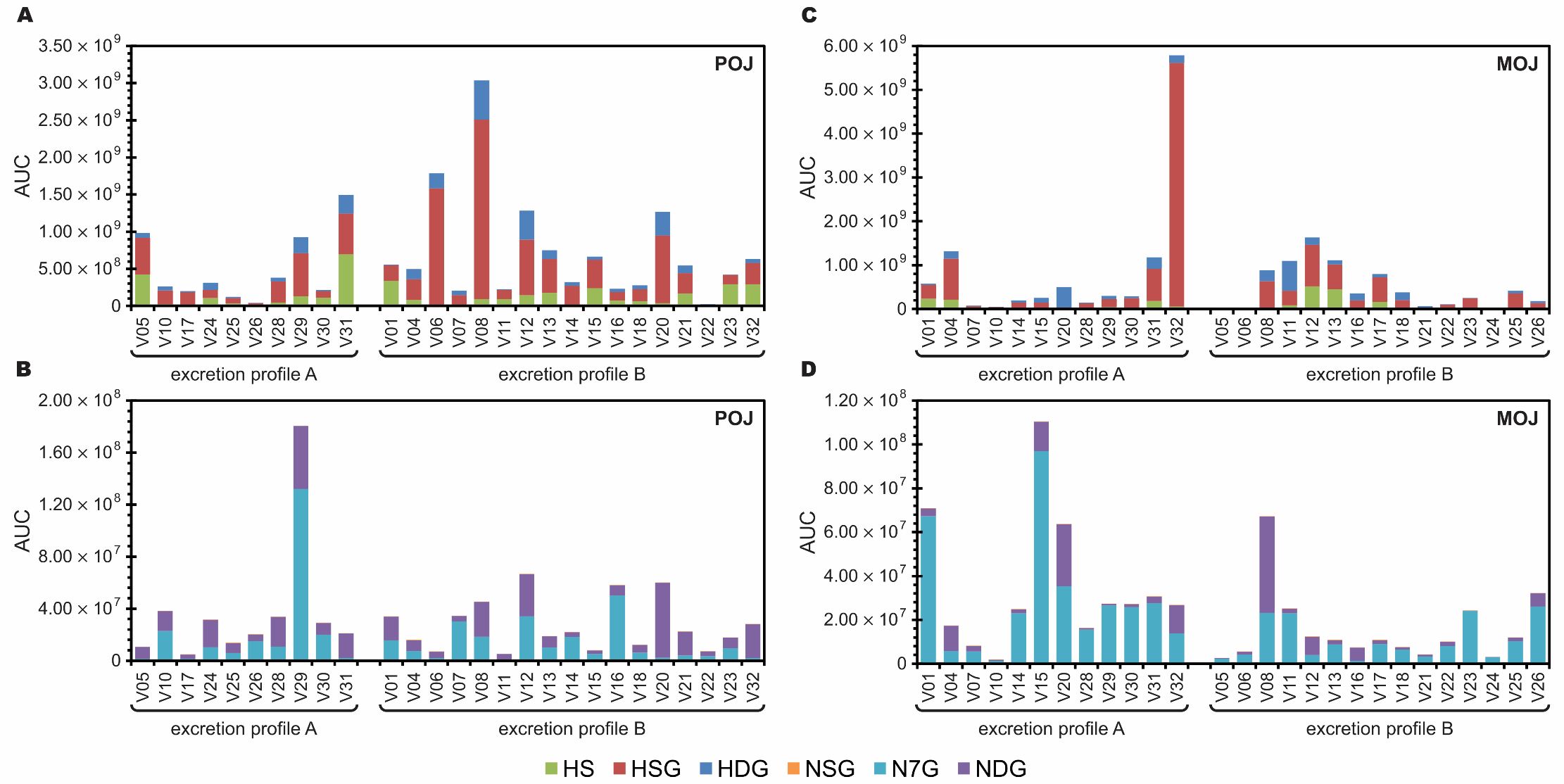


**Figure S4.** Total flavanone metabolites urinary recovered in urine over a 24-hour period for individuals classified as having Excretion Profiles A and B after consumption of Pera (POJ) and Moro (MOJ) orange juices. (A) Excretion of hesperetin metabolites after POJ consumption. (B) Excretion of naringenin metabolites after POJ consumption. (C) Excretion of hesperetin metabolites after MOJ consumption. (D) Excretion of naringenin metabolites after MOJ consumption. HS: Hesperetin-sulfate. HDG: Hesperetin-diglucuronide. HSG: Hesperetin-sulfo-*O*-glucuronide. NDG: Naringenin-diglucuronide. NSG: Naringenin-sulfo-*O*-glucuronide. N7G: Naringenin-7-*O*-glucuronide.

Reference

1. Mullen, W.; Yokota, T.; Lean, M.E.J.; Crozier, A. Analysis of ellagitannins and conjugates of ellagic acid and quercetin in raspberry fruits by LC–MSn. *Phytochemistry* **2003**, *64*, 617–624, doi:10.1016/s0031-9422(03)00281-4.
2. Hillebrand, S.; Schwarz, M.; Winterhalter, P. Characterization of Anthocyanins and Pyranoanthocyanins from Blood Orange [*Citrus sinensis* (L.) Osbeck] Juice. *J. Agric. Food Chem.* **2004**, *52*, 7331–7338, doi:10.1021/jf0487957.
