# Supplementary figures and images for: Stratification of Volunteers According to Flavanone Metabolite Excretion and Phase II Metabolism Profile after Single Doses of ‘Pera’ Orange and ‘Moro’ Blood Orange Juices
Source: Nutrients. 2021 Jan 30;13(2):473. doi: 10.3390/nu13020473 (PMC7910827; doi:10.3390/nu13020473)

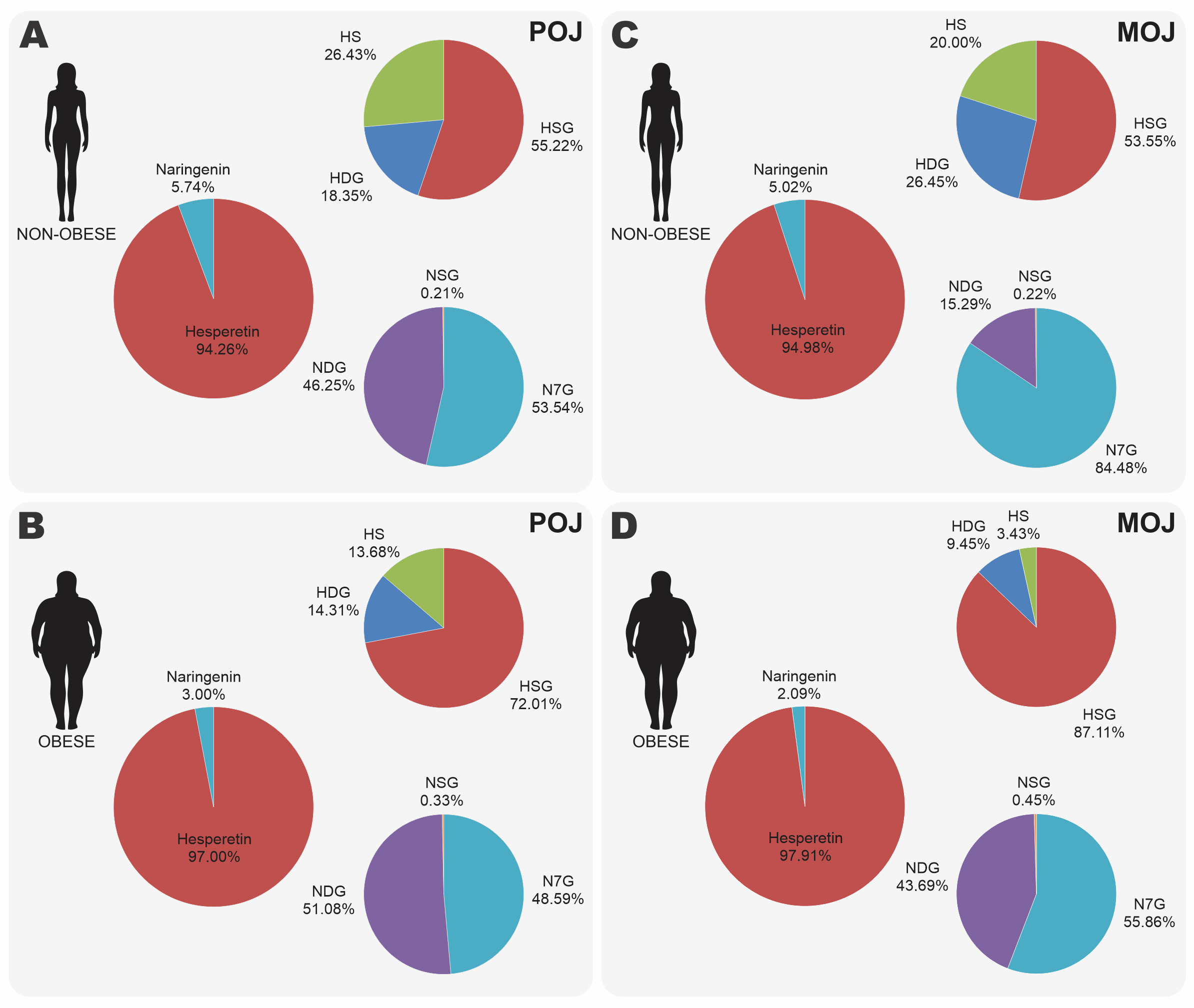

Supplement: Supplementary file 1 [file nutrients-13-00473-s001.zip › supplementary/HassimottoNMA_Figure S1.tif]

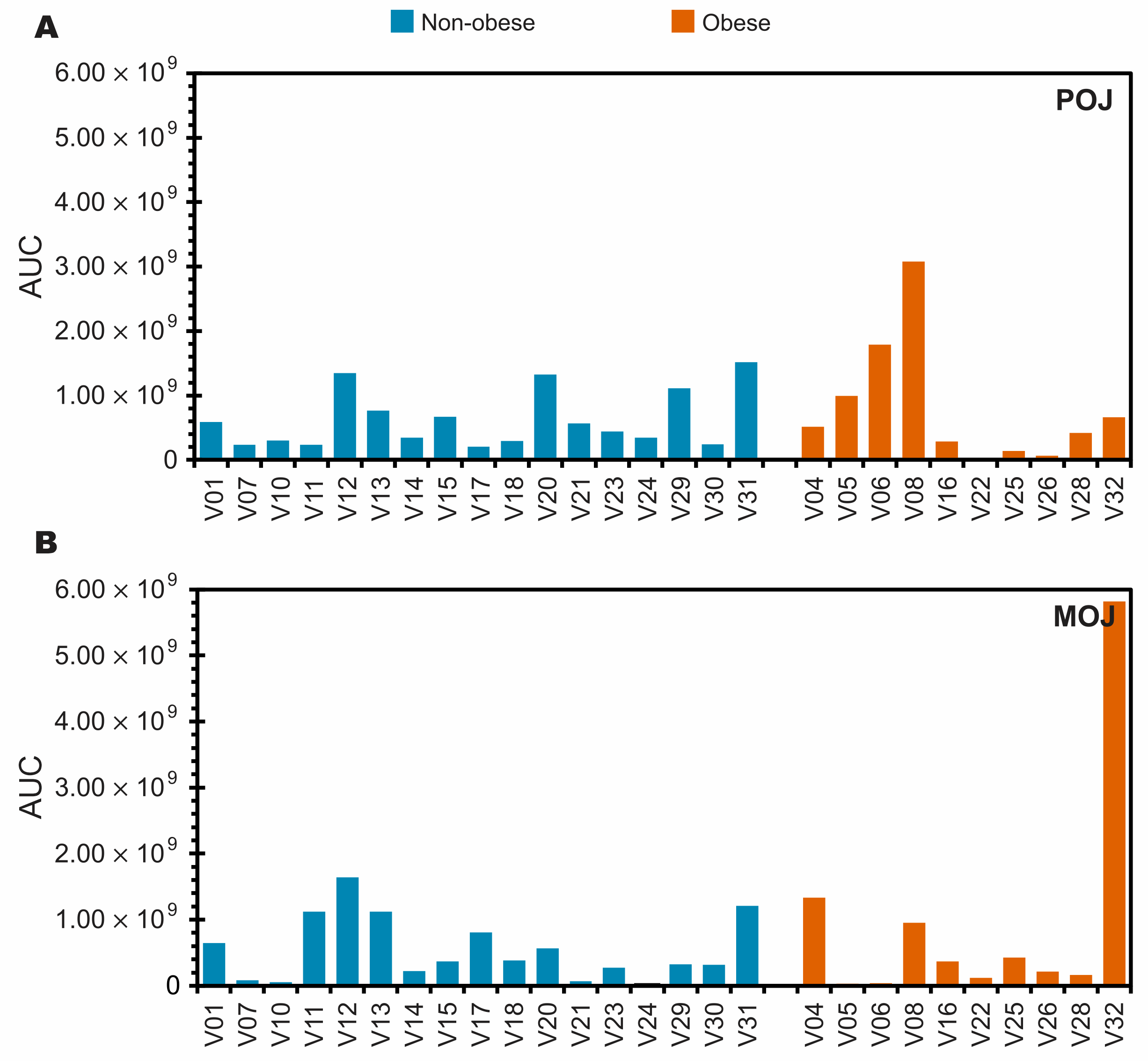

Supplement: Supplementary file 1 [file nutrients-13-00473-s001.zip › supplementary/HassimottoNMA_Figure S2.tif]

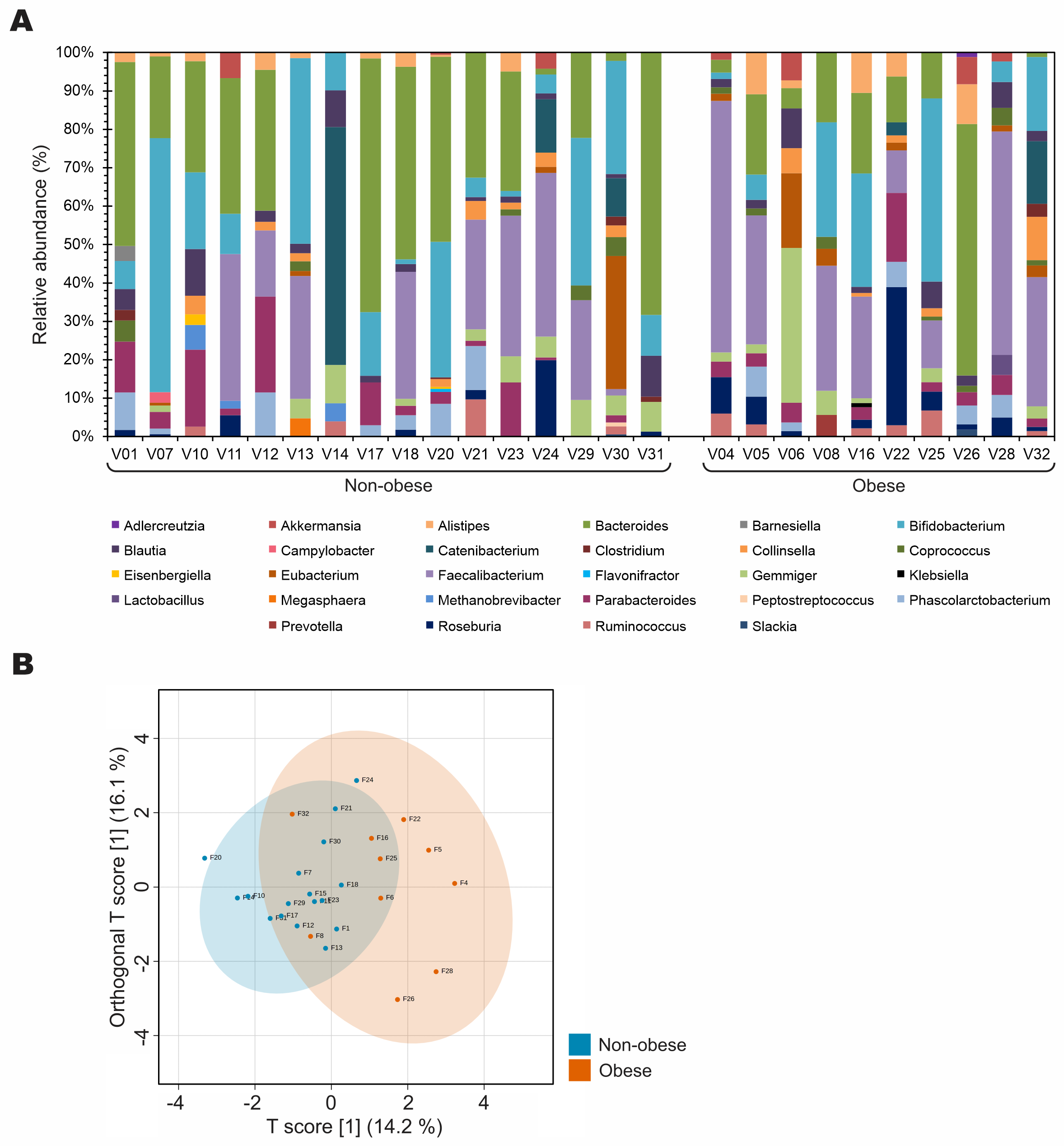

Supplement: Supplementary file 1 [file nutrients-13-00473-s001.zip › supplementary/HassimottoNMA_Figure S3.tif]

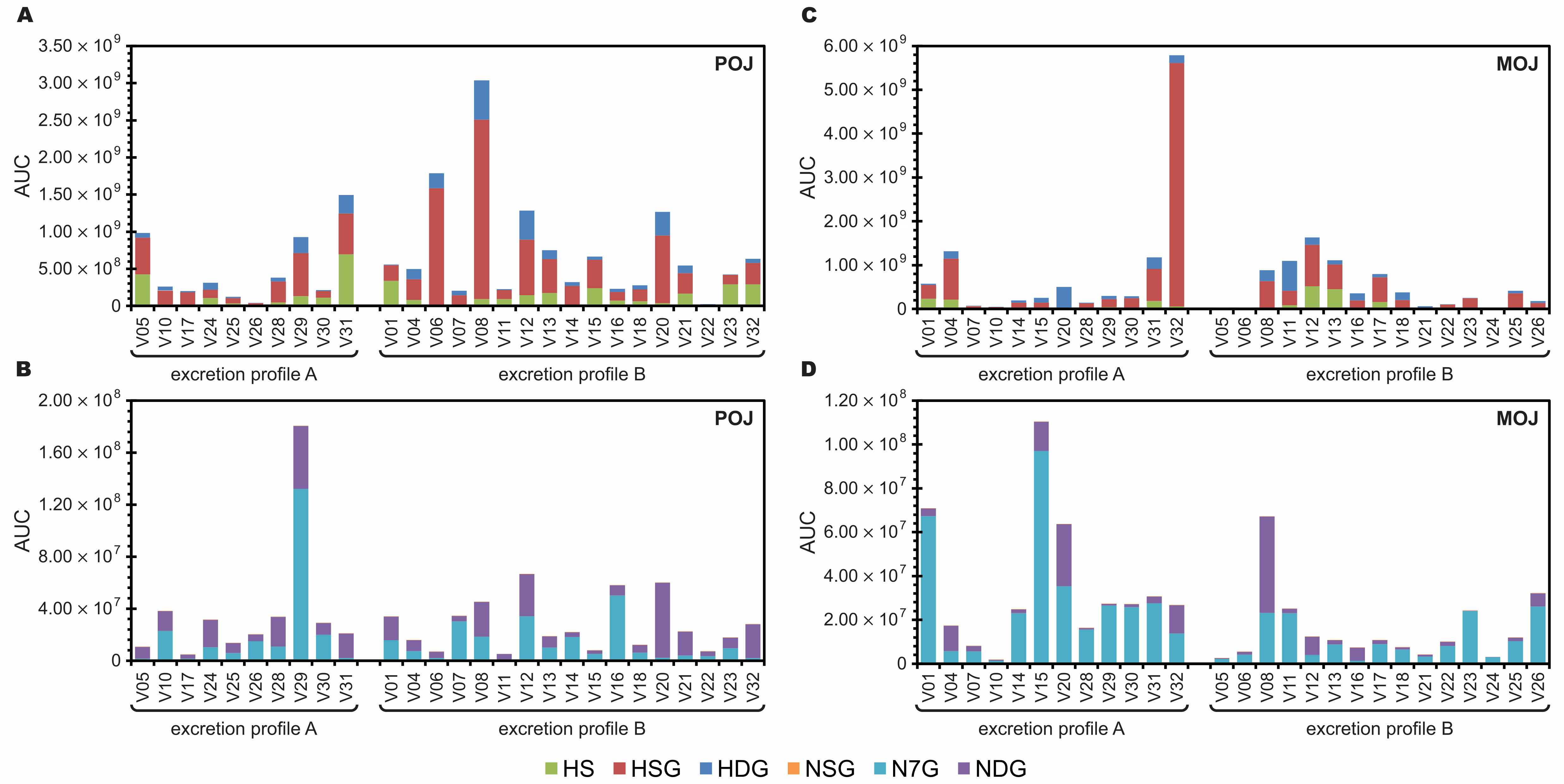

Supplement: Supplementary file 1 [file nutrients-13-00473-s001.zip › supplementary/HassimottoNMA_Figure S4.tif]
